# The Influence of Sleep and Diet on Human Peripheral Immunity and Chronic Health Conditions

**DOI:** 10.34133/research.1081

**Published:** 2026-02-19

**Authors:** Yiran Zhao, Wenran Li, Bingjie Li, Siyu Zhou, Xianlei Zhao, Qi Wang, Yingyu Cheng, Yali Luo, Jingxuan Han, Xuling Han, Helian Li, Jian Gao, Jialin Zhao, Zhonghan Sun, Mengmeng Kong, Xiaofeng Zhou, Ying Yu, Wanwan Hou, Qinsheng Chen, Jingxian Zhang, Xiaofeng Wang, Jingchun Luo, Li Jin, Leming Shi, Yan Zheng, Human Phenome Project Technological Consortium, Human Phenome Project Scientific Consortium, Huiru Tang, Sijia Wang, Feng Qian

**Affiliations:** ^1^ Human Phenome Institute, Huadong Hospital, Fudan University, Shanghai 201203, China.; ^2^ Shanghai Institute of Nutrition and Health, University of Chinese Academy of Sciences, Chinese Academy of Sciences, Shanghai 200031, China.; ^3^ State Key Laboratory of Genetics and Development of Complex Phenotypes, School of Life Sciences, Human Phenome Institute, and Zhongshan Hospital, Fudan University, Shanghai 200438, China.; ^4^ Ministry of Education Key Laboratory of Contemporary Anthropology, School of Life Sciences, Fudan University, Shanghai 200438, China.; ^5^ Institute of Immunophenome, International Human Phenome Institutes (Shanghai), Shanghai 200438, China.; ^6^ Shanghai Key Laboratory of Clinical Geriatric Medicine, Shanghai 200040, China.; ^7^ Shanghai Institute of Geriatrics and Gerontology, Shanghai 200040, China.

## Abstract

Exposures that disrupt the immune system can affect human health. This study aimed to understand immune variability influenced by exposures from the perspectives of systems biology and multiomics. We recruited 1,001 healthy participants and collected 183 exposures, 1,332 immunophenotypes, whole blood transcriptome, and plasma metabolome. Through exposure–immune wide association analysis, we identified 81 significant signals, with sleep and diet emerging as dominant exposures affecting the immunity. Sleep and diet influence the proportions of innate immune cells and the expression levels of immune cell surface proteins such as CD85j and CD16, respectively. Notably, distinct from the increase in interleukin-1β secretion caused by short-term late sleep onset, long-term late sleep onset triggered chronic inflammation with more metabolic changes. On the basis of the intracorrelation structure of exposure data, composite exposures were constructed and were found to have additional effects on immunophenotypes. Bidirectional mediation analysis revealed that sleep effects on immunity are commonly linked to the transcriptome, whereas dietary influences on immunity are primarily associated with the metabolome. We quantified the mediation effects of exposures, omics, and immunophenotypes and further demonstrated that these effects reflect human immune health or chronic diseases. Our study drew a comprehensive map of “exposure–immunome–omics” and is expected to provide guidance for future health assessment and management.

## Introduction

The vital role of the immune system in maintaining homeostasis makes it highly susceptible to various disturbances, extending beyond pathogens to environmental and lifestyle exposures. These exposures are thought to contribute to disease development by altering the internal environment, which acts as a biological signal reflecting external conditions and disease progression [1–3]. Existing studies of such projects as the Milieu Intérieur project [4], the Stanford 1,000 Immunomes Project [5], and the Human Functional Genomics Project (500FG) [6,7] have demonstrated the fundamental role of exposures in immune system variation [4–9]. Research by Brodin et al. [5] and Carr et al. [8] has revealed that nonheritable factors are the primary drivers of immune system changes. The EXIMIOUS project [10], conducted under the European Union’s Horizon 2020 program, has also sought to map exposure-induced changes of immunome (immunophenotypes, which include the proportion of immune cells and their functions, such as the expression of immune-related proteins and the capacity for cytokine secretion). However, prior investigations have been constrained by limited coverage of exposure or immunophenotypic data, focusing mainly on specific factors such as vaccination status [8] and smoking [4,9]. Consequently, the broader spectrum of immunophenotypes influenced by various exposures needs more attention [11], especially in East Asian populations.

As a central regulator of the human body’s internal environment, the immune system interacts with diverse biomolecules to maintain overall health. Various biomarkers, including immunophenotypes, offer mechanistic insights into how exposures affect health. However, research that systematically explores the molecular mediators between exposure and immunity remains limited and has focused primarily on smoking [9]. Saint-André et al. [9] recently used epigenetic and proteomic data to elucidate the role of omics in smoking-induced immune alterations. Comprehensive investigations are needed to identify additional molecules that are associated with exposure-induced immune responses. Moreover, the potential of these molecules as indicators of health status also remains largely underexplored.

In this study, we profiled 1,332 immunophenotypes across 4 categories, 183 exposures across 12 categories, 12 composite exposures, and multiomics data (transcriptome and metabolome) in The Human Phenome Atlas (THPA) cohort of 1,001 healthy East Asian individuals aged 20 to 60 years. Through systematic association analyses, we identified 55 immunophenotypes that were influenced by 20 single or 3 composite exposures. Our bidirectional mediation analysis revealed that specific genes, lipids, and metabolites serve as mediators or outcomes linking exposures to immune signatures. In addition, we developed transcriptomic (T-) and metabolomic (M-) indexes by summing weighted scores of molecules in mediation linkages. These indexes can reflect both exposure and immune status through omics. We subsequently evaluated their association with immune health and chronic diseases across 8 external cohorts. In summary, our study systemically charted a map of exposure–immunophenotype associations, highlighting transcriptional and metabolic molecules that interact with immunophenotypes under exposure influences and their potential implications for human health.

## Results

### Immunome in THPA cohort

**Fig. 1.**
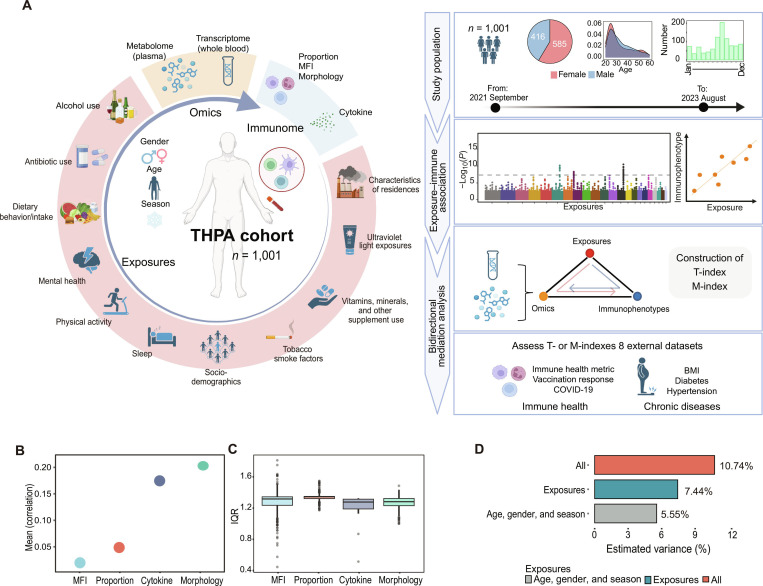
Overview of the data and study design. (A) All the types of data that have been analyzed for this study (left) and population characteristics and analysis overview (right). Created with BioRender.com. (B and C) Mean of different category immunophenotypes’ correlations (B); boxplot of different category immunophenotypes’ IQRs (C). (D) Bar plot showing the interindividual variation in the immunome (gender, age, and season are modulators of the immunophenotypes) explained by the indicated exposures in multivariate PERMANOVA analysis. All, all of the indicated factors combined.

THPA cohort includes 416 men and 585 women from 20 years of age to 60 years of age. In THPA cohort, we profiled 4 categories of immunophenotypes, comprising a total of 1,332 phenotypes: 327 proportion data, 684 mean fluorescence intensity (MFI) data reflecting immune cell surface protein expression (Fig. S2), 302 morphology data, and 19 cytokine data including plasma cytokines and stimulated cytokine levels (Fig. 1A and Table S1). The immunophenotypic data reflect the peripheral immune status at multiple levels. Intracorrelation analysis revealed that the morphology data exhibited the highest internal correlation, followed by the cytokine, proportion, and MFI data. The MFI data also showed the largest interquartile range (IQR; Fig. 1B and C), indicating that there was greatest variability within immune cell status. These results revealed that different immunological data types had distinct degrees of internal correlation, underscoring the complexity of immunome variation.

### Exposures explaining interindividual immunome variations

In this study, we also collected data on 183 exposures using self-reported questionnaires. These exposures can be classified into 12 categories: “alcohol use”, “antibiotic use”, “characteristics of residences”, “dietary behavior”, “dietary intake”, “mental health”, “physical activity”, “sleep”, “sociodemographics”, “tobacco smoke factors”, “ultraviolet light exposures”, and “vitamins, minerals, and other supplement use” (Fig. 1A and Table S2).

We first systematically examined the associations between immunophenotypes and basic factors such as age, gender, and season. We found that more than half of the immunophenotypes were significantly influenced by these factors: 56.9% were correlated with age, 56.1% with gender, and 31.5% with season (false discovery rate [FDR] < 0.05; Table S1). To assess the impact of exposures in explaining interindividual immunome variability, we calculated the proportion of variance explained by basic factors and exposures for the whole immunome profile. The results showed that exposures explained 7.44% of the variance in immunome, while age, gender, and season collectively accounted for 5.55%, consistent with previous reports [11]. Together, these factors explained 10.74% of the variance in the overall immune profile (FDR < 0.05; Fig. 1D). When examining the variation across different immune categories, exposures explained 9.95% of the variation in proportion data, 6.74% in MFI data, 4.24% in morphology data, and 3.19% in cytokine data (Fig. S3A). Exposures had a greater effect on innate immunophenotypes in the proportion data, whereas they had a stronger influence on adaptive immunophenotypes in the MFI data (Fig. S3B).

To identify the specific exposures that contributes the most to the variability in immunome, we adjusted the immunophenotypes for age, gender, and season. We found that the unhealthy plant-based diet index (uPDI), the Morningness–Eveningness Questionnaire (MEQ) score (also termed the “chronotype”; low MEQ score represents “evening chronotype”), and fresh vegetables intake were the exposures that contributed the most to the variation in the immunome (Fig. S3C). Of these, MEQ score accounted for the highest variance in innate immunity within the proportion data (Fig. S4A). MEQ score and uPDI also explained the most variance in adaptive immunity within the MFI data (Fig. S4D). In addition, black tea intake was the exposure that explained the most variance in adaptive immunity within the proportion data (Fig. S4B), while coffee consumption per day was the top exposure influencing innate immunity within the MFI data (Fig. S4C). All these top-ranked exposures are related to sleep or diet.

### Sleep and diet as dominant exposures shaping the human immunome

**Fig. 2.**
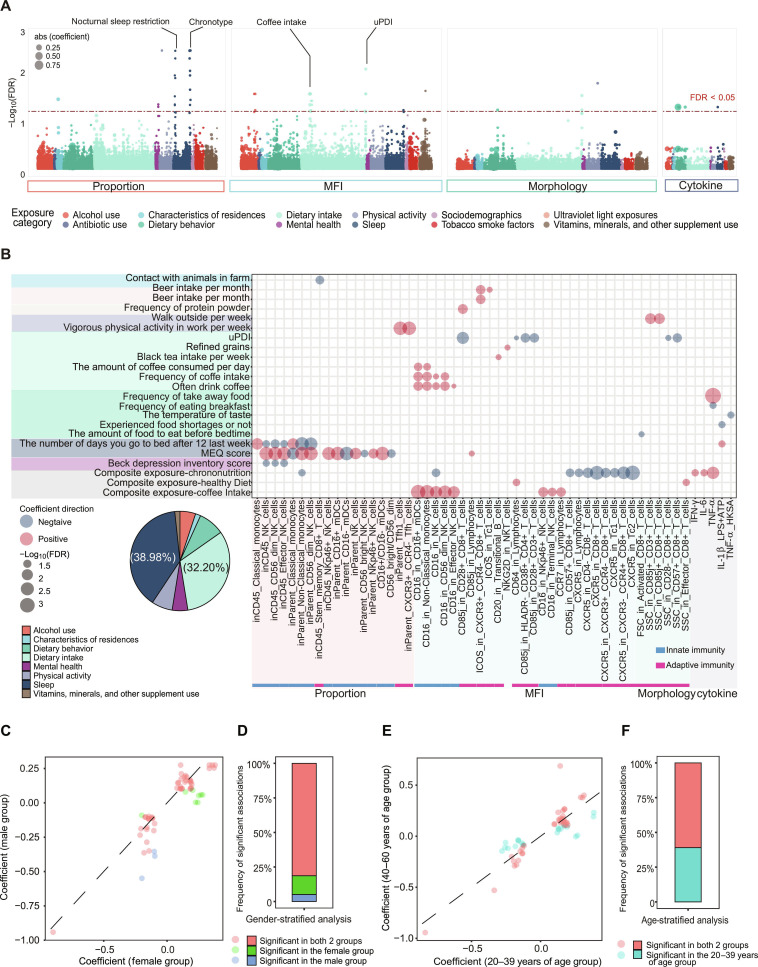
Associations between immunophenotypes and single/composite exposures. (A) Summary of the associations between single exposures and different categories of immunophenotypes. The *y* axis indicates the −log_10_(FDR) of the regression coefficient for each of the association between exposure and immunophenotype. The colors represent different exposure categories. Circles larger than the dashed line represent significant associations with FDR < 0.05. abs, absolute. (B) Bubble plot of the significant associations between single (or composite) exposures and different categories of immunophenotypes. The size of circles represents −log_10_(FDR) of the regression coefficient for each of the association between exposure and immunophenotype. Red represents a positive association between exposure and immunophenotype; blue represents a negative association between exposure and immunophenotype. Pie chart shows the proportion of different exposure categories in bubble plot. (C to F) Dot plot showing the coefficient of significant associations in (A) in the stratified analysis by gender (C) or age (E). Dashed lines in the plots are diagonal lines. Bar plot showing the proportion of significant associations in (A) in the gender (D) or age (F) stratified analysis. In (C) to (F), participants are divided into different groups based on gender and age: female (*n* = 585) and male (*n* = 416) groups and 20 to 39 years of age (*n* = 737) and 40 to 60 years of age (*n* = 264) groups.

The comparison of the relative importance of different exposures in explaining immunome variation has shown that diet and sleep are the most crucial category of exposures. Further, to identify individual immunophenotypes influenced by these exposures, we tested pairwise associations between 1,332 immunophenotypes and 183 exposures using multiple linear regression models. The analysis controlled for age, gender, and season, with significance determined by an FDR of <0.05 for both the coefficient and the likelihood ratio test (LRT). A total of 59 significant associations were identified: 27 in the proportion data, 22 in MFI, 5 in morphology, and 5 in cytokines (Fig. 2A and Table S3). Notably, 38.98% of these associations were linked to sleep-related exposures, whereas 32.20% were linked to diet-related exposures (Fig. 2B), suggesting again that sleep and diet are the most influential exposure categories affecting immunophenotypes.

Among all the significant associations, sleep-related exposures primarily influenced the proportions of innate immune cells (Fig. 2A and B). For example, chronotype, as assessed by the MEQ score, was positively associated with the proportions of natural killer (NK) cell subsets (excluding CD56^bright^ NK cells; range of coefficients, −0.170 to 0.147; FDR of coefficients < 0.05) and nonclassical monocytes (coefficient = 0.156; FDR of coefficient = 0.020). In contrast, nocturnal sleep restriction, as assessed by the question “the number of days you go to bed after 12 last week”, exhibited an opposing relationship (Fig. 2B).

In addition, dietary exposures mainly affected the expression level of immune cell surface proteins (Fig. 2A and B). For instance, the uPDI was negatively correlated with CD85j expression on T cell subsets (range of coefficients, −0.251 to −0.224; FDR of coefficients < 0.05), while frequency and amount of coffee consumption were positively correlated with CD16 expression on innate immune cells (range of coefficients, 0.101 to 0.335; FDR of coefficients < 0.05; Fig. 2B). CD85j is a negative regulatory receptor, related to aging and cytomegalovirus infection [12].

### Long-term and short-term late sleep onsets lead to distinct inflammatory states

Association analysis revealed a marked overlap in the immunophenotypes affected by sleep-related exposures. To further dissect the effects of evening chronotype (“long-term late sleep onset”) and nocturnal sleep restriction (“short-term late sleep onset”) on immunity, we performed a stratified analysis based on chronotype (see the “Stratified analysis of MEQ score” section and Fig. S5A). This analysis demonstrated that nocturnal sleep restriction induced an acute inflammatory state in individuals with a middle chronotype (“normal biological clock” or “normal sleep onset”), characterized by an increased proportion of classical monocytes and interleukin-1β (IL-1β) secretion after lipopolysaccharide (LPS) and adenosine triphosphate (ATP) stimulation (Nod-like receptor family pyrin domain-containing 3 [NLRP3] inflammasome; Fig. S5B), whereas individuals with an evening chronotype exhibited a shift toward chronic inflammation, characterized by reduced CD85j expression and an unbalanced ratio of monocyte and myeloid dendritic cell (mDC) subpopulations (Fig. S5C).

### Robustness and sensitivity of the exposure–immune associations

To demonstrate the robustness of the exposure effects on immunophenotypes, we conducted a resampling analysis within THPA cohort. We randomly selected 90% (Fig. S6A) or 80% (Fig. S6B) of the total samples and tested the exposure effects on immunophenotypes. The process was repeated 100 times, and we observed that all of 59 significant associations remained consistent with the original full dataset in more than 80% of the sampling iterations (Table S4). In addition, we further performed sensitivity analyses on these 59 significant associations to account for potential confounders, including the first 20 genetic principal components (PCs), body mass index (BMI), and coronavirus disease 2019 (COVID-19) or COVID-19 vaccination status (Table S5). All the associations remained significant after adjustment for each of these confounders individually.

To explore the exposure–immune associations across subgroups defined by gender or age, we conducted stratified analyses. Among the 59 associations, more than half were significant in both subgroups, and all had the same effect direction in both subgroups (Fig. 2C to F and Table S6). In addition, there were several gender- or age-specific associations between exposure and immune (Table S7). For example, we observed that income-related changes in immune cell proportions were only detectable in individuals aged 20 to 39 years. Higher income was associated with an increased proportion of effector T cells (range of coefficients, 0.073 to 0.106; FDR of coefficients < 0.05) and a decreased proportion of suppressor T cells, such as naïve T regulatory cells (range of coefficients, −0.129 to −0.088; FDR of coefficients < 0.05).

### Composite exposures have additional effects on immunophenotypes

Previous studies have shown that exposures within populations often exhibit patterns of correlation [13,14]. Health status is seldom attributable to a single exposure but rather to a combination of factors. To address this, we introduced “composite exposures”, which extracted shared information from sets of exposures with high correlations (Spearman correlation’s coefficient > 0.2, Bonferroni < 0.05). These sets of exposures were obtained through unsupervised Ward clustering and represented the combined exposure habits of the population. Then, using a partial least-squares path model, we identified 12 composite exposures (latent variables 1 to 12 [LV1 to LV12]) in THPA cohort, which were uncorrelated with each other (Fig. S7A and Table S8). To ensure the robustness of these findings, we randomized 80% of the sample and repeated this analysis, consistently identifying almost identical 12 composite exposures.

We conducted association analyses between these composite exposures and immunophenotypes and identified 22 significant associations (FDR of coefficient < 0.05; FDR of LRT < 0.05; Fig. S7B and Table S9). Among the 12 composite exposures, LV8 to LV10 were significantly associated with specific immunophenotypes. On the basis of their compositions and the loading of the compositions, we termed LV8 as “coffee intake”, LV9 as “healthy diet”, and LV10 as “chrononutrition” [15,16] (Fig. S7C). A higher chrononutrition score indicates more delayed eating and sleeping times. Coffee intake was positively correlated with CD16 expression on innate immune cells (Fig. 2B), which is consistent with the results of single exposure analysis. Moreover, there were 65.22% of the immunophenotypes independently identified in the composite exposure analysis (Fig. S7D). For example, chrononutrition was negatively associated with CXCR5 expression on T cell subsets (range of coefficients, −0.177 to −0.143; FDR of coefficients < 0.05) and positively associated with levels of inflammatory cytokines, including interferon-γ (IFN-γ), IL-6, and tumor necrosis factor-α (TNF-α) (range of coefficients, 0.122 to 0.153; FDR of coefficients < 0.05; Fig. 2B). CXCR5^+^CD8^+^ T cells have been implicated in viral infections, autoimmunity, and antitumor responses [17,18]. These findings highlight the added value of this approach in uncovering previously unrecognized effects of combined exposures on immunophenotypes.

### Sleep and diet affect different immune-related biological processes

**Fig. 3.**
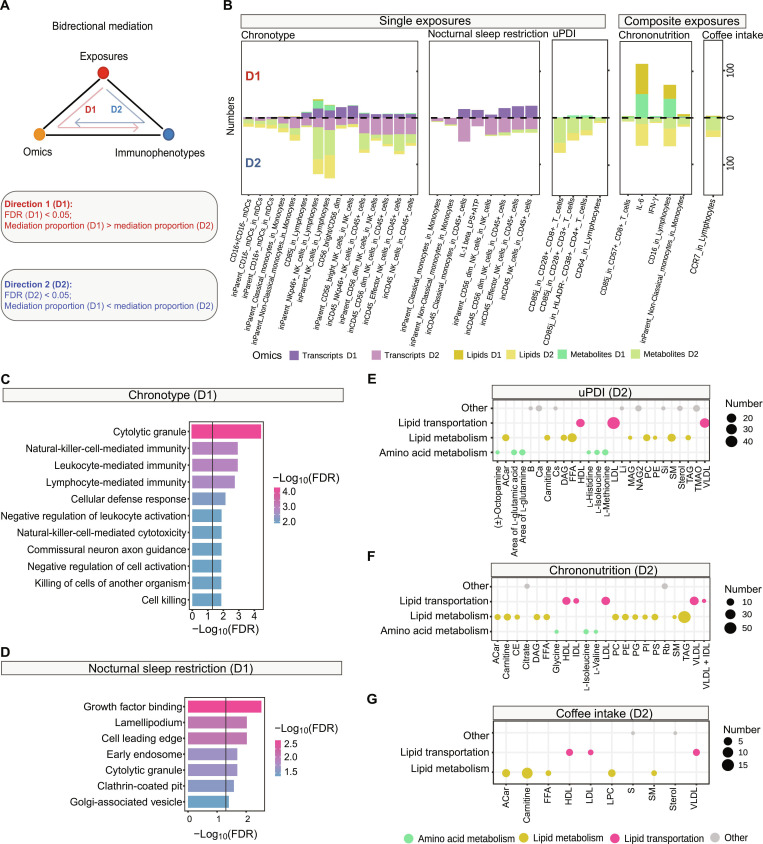
Bidirectional mediation analysis of exposures, omics, and immunophenotypes. (A) Framework for bidirectional mediation analysis among single or composite exposures, omics, and immunophenotypes. D1 mediation means exposures influence omics-mediated immunophenotypes, being selected by FDR (D1) < 0.05 and mediation proportion (D1) > mediation proportion (D2); D2 mediation means exposures influence omics mediated by immunophenotypes, being selected by FDR (D2) < 0.05 and mediation proportion (D2) > mediation proportion (D1). (B) Bar plots showing the number of the D1 and D2 mediations between omics and immunophenotypes (FDR of ACME < 0.05). Each panel represents the result of the mediation analysis of a specific exposure. Only the results of exposures that have mediation relationships with more than 10 molecules are shown in the plot. (C and D) Enriched GO pathways of genes that have significant ACME in D1 mediations of chronotype (C) and nocturnal sleep restriction (D). (E to G) Classification of lipids and metabolites that have significant ACME in D2 mediations of uPDI (E), chrononutrition (F), and coffee intake (G). The size of bubble in the plot means the number of lipids or metabolites in each metabolome group. They were categorized into 4 pathways: lipid transport, lipid metabolism, amino acid metabolism, and other. ACar, acyl carnitine; B, boron; Ca, calcium; Cs, cesium; DAG, diacylglycerol; FFA, free fatty acids; HDL, high-density lipoprotein; LDL, low-density lipoprotein; Li, lithium; MAG, monoacylglycerol; NAG2, *N*-acetyl groups of *N*-acetyl-glycoproteins (*N*-acetyl groups of *N*-acetylneuraminic acid); PC, phosphatidylcholine; PE, phosphatidylethanolamine; Si, silicon; SM, sphingomyelin; TAG, triacylglycerol; TMAO, trimethylamine *N*-oxide; VLDL, very-low-density lipoprotein; CE, cholesterylesters; IDL, intermediate density lipoprotein; PG, phosphatidylglycerol; PI, phosphatidylinositol; PS, phosphatidylserine; Rb, rubidium; LPC, lysophosphatidylcholine; S, sulfur.

The changes in peripheral blood immune cells following external perturbations are an integrated process, involving orchestrated alterations with other molecules and ultimately driving broader health consequences. To explore the molecular biological pathways through which exposures affect immunity, we conducted a bidirectional mediation analysis. This analysis evaluated the role of molecules (transcriptome and metabolome) in the relationships between exposures and immunophenotypes (Fig. 3A). The analysis identified 1,946 mediation linkages (Tables S10 to S13): 533 represented the effect of exposures on immunophenotypes mediated by molecular mediators (direction 1 [D1]), while 1,413 represented the effect of exposures on molecules mediated by immunophenotypes (D2). Summarizing these linkages, we observed distinct patterns for sleep and diet in their influence on molecules and immunophenotypes.

Sleep (e.g., chronotype and nocturnal sleep restriction) was shown to influence immunophenotypes primarily through interactions with the transcriptome (Fig. 3B). Compared to nocturnal sleep restriction, chronotype also led to changes in the metabolome, illustrating that long-term sleep habits (“chronotype”) have a greater impact on peripheral blood health status than short-term late sleep onset (“nocturnal sleep restriction”). Gene Ontology (GO) enrichment analysis revealed that long-term sleep habits and short-term late sleep onset not only affect different immune states but also involve different biological processes (Fig. 3C and D). Genes involved in the effect of chronotype on innate immunophenotypes were enriched in pathways associated with cytolytic granules and NK-cell-mediated immunity (Fig. 3C and Fig. S9A). In contrast, genes mediating the effect of nocturnal sleep restriction on immunophenotypes were predominantly enriched in pathways related to growth factor binding, lamellipodium formation, and the cell leading edge (Fig. 3D).

Contrary to sleep, diet (e.g., uPDI, coffee intake, and chrononutrition) mainly affected lipid and metabolite profiles mediated by immunophenotypes (Fig. 3B). The exception is that chrononutrition was linked to IL-6 secretion and CD16 expression on lymphocytes through several lipids and metabolites. We categorized all the lipids and metabolites involved in the mediation linkages into 4 groups based on their biological implication: “lipid transportation”, “lipid metabolism”, “amino acid metabolism”, and “other”. Among them, lipid transportation and lipid metabolism encompass the majority of lipids and metabolites related to diet, sleep, and immunity (Fig. 3E to G and Fig. S9B and C). In addition, uPDI, chrononutrition, and chronotype were further associated with changes in amino acid metabolism (Fig. 3E and F and Fig. S9B and C). Notably, amino acids were also found to be highly correlated with chronotype in the prior study [19]. Our finding further expands the understanding of the role of these amino acids in the relationship between chronotype and immunophenotypes.

### Genes linked to sleep reflect immune health

**Fig. 4.**
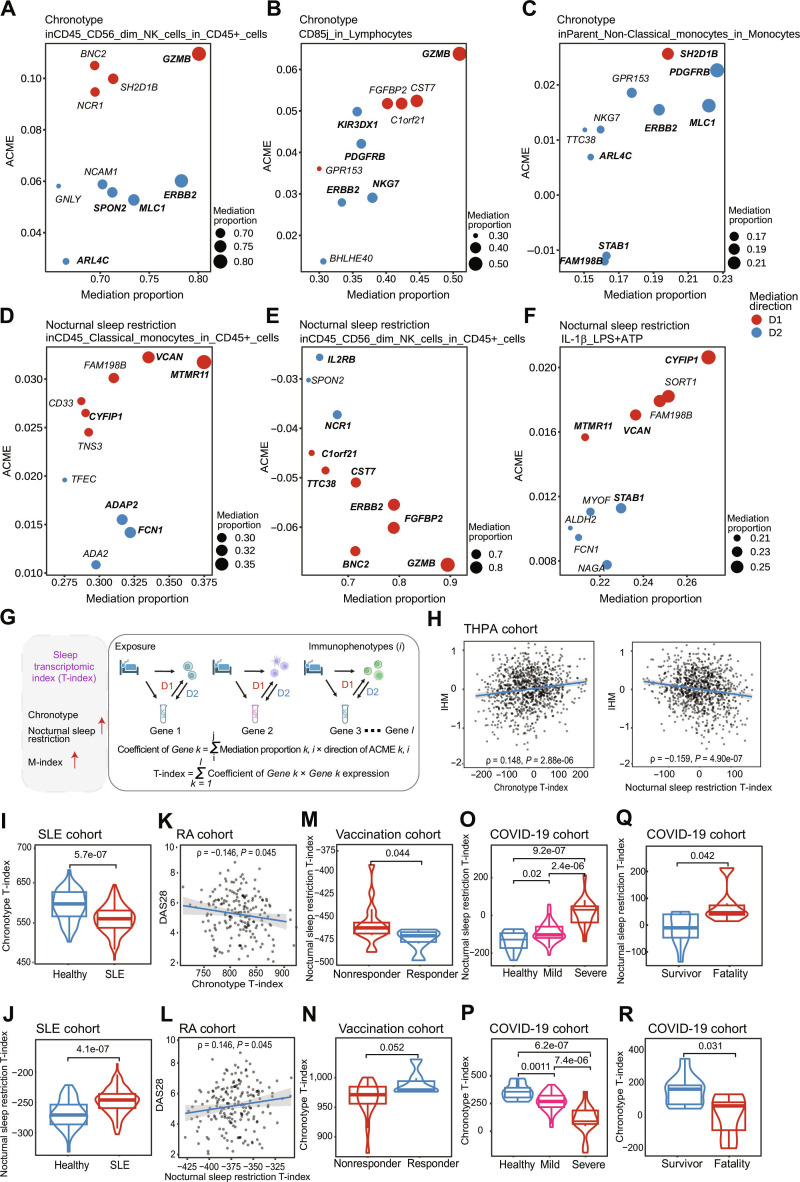
Top-ranked genes in the mediation analysis and T-index relationships with immune health. (A to F) The estimated proportion and ACME of mediation for the top 10 ranked genes (according to mediation proportions) on the associations of chronotype (A to C) or nocturnal sleep restriction (D to F) with immunophenotypes (for each exposure, only the results of the top 3 immunophenotypes with mediation relationships are shown). Subtitles of the graph represent exposure and immunophenotype in the mediation linkage. The *y* axis represents the ACME of mediation, the *x* axis and the size of the circle represent the proportion of mediation, and the color of the circle represents the direction of mediation (red, D1; blue, D2). Top-ranked genes used to calculate the T-indexes are in bold. (G) A schematic illustration of the construction of the weighted sum of gene expressions. The weighted sum of the bidirectional mediation proportions for each gene was calculated to determine its coefficient. These coefficients were then used as weights to calculate the weighted sum of all gene expression levels, which served as the index. (H) Scatter plot with trend line showing the positive correlation between chronotype T-index and IHM [23] and the negative correlation between nocturnal sleep restriction and IHM [23]. Pearson’s correlation and associated *P* value are shown. The shaded area represents the 95% confidence interval (95% CI). (I and J) Chronotype (I) or nocturnal sleep restriction (J) T-index stratified by SLE in the pediatric SLE cohort [25]. Averaged indexes were used for patients with multiple visits. *P* values from paired, 2-tailed Wilcoxon test are shown (*n* = 46 healthy and 158 SLE). (K and L) Correlation between baseline (pretreatment) chronotype (K) or nocturnal sleep restriction (L) T-index and DAS28 of 190 patients with RA in the RA-MAP study [26]. Pearson’s correlation and associated *P* value are shown. The shaded area represents the 95% CI. (M and N) Chronotype (M) or nocturnal sleep restriction (N) T-index before influenza vaccination in elderly stratified by their response status [29] (*n* = 6 responders and 17 nonresponders). *P* values from paired, 2-tailed Wilcoxon test are shown. (O and P) nocturnal sleep restriction T-index (O) and chronotype T-index (P) stratified by disease severity of COVID-19 in the COVID-19 cohort [30]. Averaged indexes were used for patients with multiple visits. *P* values from paired, 2-tailed Wilcoxon test are shown (*n* = 14 healthy, 50 mild, and 16 severe). (Q and R) Nocturnal sleep restriction T-index (Q) and chronotype T-index (R) stratified by outcome of severe patients in the COVID-19 cohort [30]. Averaged indexes were used for patients having multiple visits. *P* values from paired, 2-tailed Wilcoxon test are shown (*n* = 10 alive and 6 died). DAS28, disease activity score in 28 joints.

Among the genes in mediation analysis, *GZMB* (granzyme B), a hallmark gene of NK cells associated with cytotoxic function [20], emerged as a top mediator linking sleep (including chronotype and nocturnal sleep restriction) to NK cell subsets (mediation proportion = 50.96% to 90.80%; Fig. 4A to E and Table S10). *GZMB* also mediated the relationship between chronotype and CD85j expression on lymphocytes (average causal mediation effect [ACME] = 0.064; mediation proportion = 50.96%; Fig. 4B). Furthermore, *NCR1*(*NKp46*) [21], *CST7* [22], and *IL2RB* [22] are all characteristic genes of NK cells and associated with sleep (ACMEs = −0.037, −0.051, and −0.026; mediation proportions = 67.9%, 71.5%, and 64.4%; Fig. 4E). For other immunophenotypes, such as monocyte proportion and ATP/LPS-stimulated IL-1β secretion, genes with different functions played a role (Fig. 4C, D, and F and Table S10). For instance, *CYFIP1* (cytoplasmic fragile X messenger ribonucleoprotein 1 interacting protein 1), as the most predominant mediator of nocturnal sleep restriction and IL-1β secretion (ACME = 0.021; mediation proportion = 26.99%; Fig. 4F), was associated with the “cell leading edge” and “lamellipodium” pathways.

To capture the interaction effect of immune and sleep-influenced gene expression, we calculated T-indexes by summing the weighted scores of the identified genes in the mediation linkages (Fig. 4G). Most of the above genes mentioned are among the top-ranked components used to construct the T-indexes (Fig. S10A and B and Table S14). In THPA cohort, we compared these T-indexes with the immune health metric (IHM) transcriptional surrogate signature score, a unified metric of overall immune health developed using a monogenic cohort in 2024 [23]. Our analysis revealed that the IHM transcriptional surrogate signature score was positively correlated with the chronotype T-index (Pearson’s correlation coefficient = 0.148, *P* = 2.88 × 10^−6^) and negatively correlated with the nocturnal sleep restriction T-index (Pearson’s correlation coefficient = −0.159, *P* = 4.90 × 10^−7^; Fig. 4H), linking evening chronotype and nocturnal sleep restriction to diminished immune health.

Nocturnal sleep restriction has been reported to promote the progression of certain autoimmune diseases [24]. To explore this relationship, we first utilized a pediatric systemic lupus erythematosus (SLE) transcriptomic dataset [25] and compared sleep-related T-indexes between 46 healthy controls and 158 patients with SLE. The analysis revealed that patients with SLE had a significantly lower chronotype T-index (2-tailed Wilcoxon test, *P* = 5.7 × 10^−7^; Fig. 4I) and a higher nocturnal sleep restriction T-index (2-tailed Wilcoxon test, *P* = 4.1 × 10^−7^; Fig. 4J). Next, we calculated sleep-related T-indexes in a rheumatoid arthritis (RA) cohort [26]. Correlation analysis demonstrated that these indexes, calculated from the baseline (pretreatment) transcriptome, were significantly associated with disease activity (Fig. 4K and L) and C-reactive protein (CRP) levels, independent of treatment effect (Fig. S12A and B and Table S18). These findings suggest a positive relationship between the late sleep onset and the progression of autoimmune diseases such as SLE and RA.

In addition, sleep habits are strongly associated with both vaccination responses [27] and the severity of COVID-19 [28]. We measured the sleep-related T-indexes before influenza vaccination in a vaccination cohort [29] and observed that the nocturnal sleep restriction T-index was higher in the elderly individuals who had no antibody response to vaccination (2-tailed Wilcoxon test, *P* = 0.044; Fig. 4M). Conversely, the chronotype T-index was relatively lower, although this test was not significant (Fig. 4N). Finally, in a COVID-19 cohort [30], we evaluated whether sleep-related T-indexes could distinguish COVID-19 disease severity. The indexes exhibited a gradient across the healthy, mild, and severe groups, with significant differences among all groups (Fig. 4O and P). Furthermore, these indexes were able to reflect the disease outcome in the severe group (Fig. 4Q and R). Survivors had a lower nocturnal sleep restriction T-index (2-tailed Wilcoxon test, *P* = 0.042; Fig. 4Q) and a higher chronotype T-index (2-tailed Wilcoxon test, *P* = 0.031; Fig. 4R) compared to the fatality group. These results underscore the critical relationships between the sleep-related T-indexes and both elderly vaccine efficacy and COVID-19 severity or outcomes, further highlighting the importance of sleep in maintaining immune health.

### Metabolites linked to sleep and diet reflect chronic diseases

**Fig. 5.**
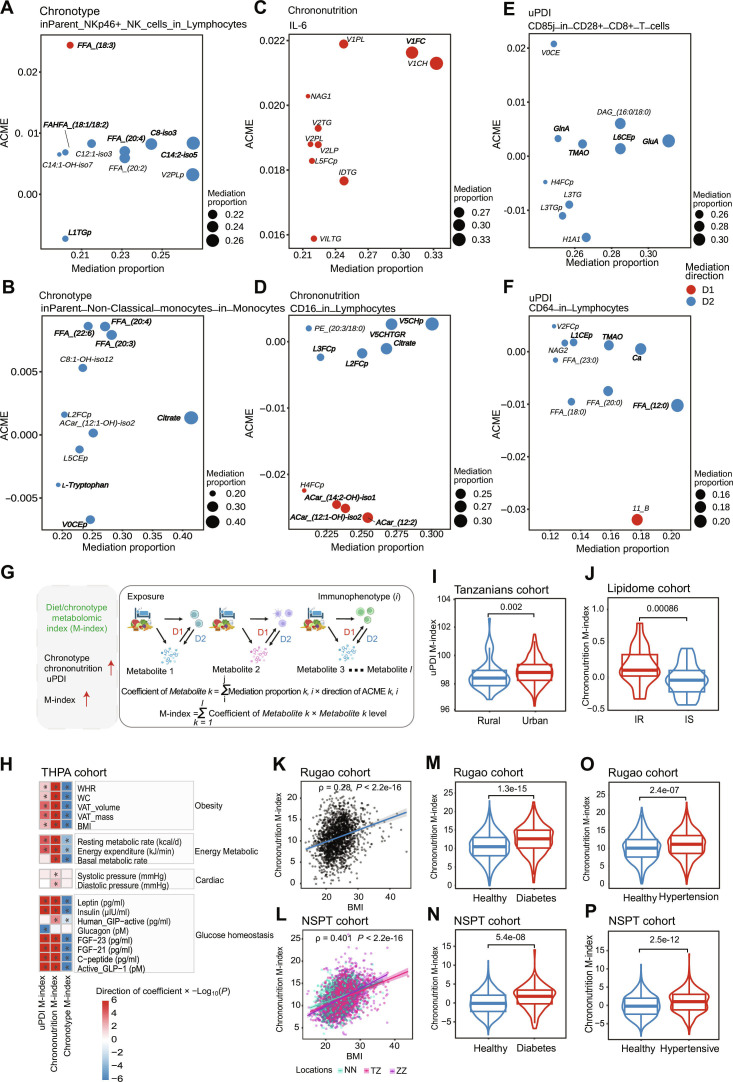
Top-ranked lipids/metabolites in the mediation analysis and M-index relationships with chronic diseases. (A to F) The estimated proportion and ACME of mediation for the top 10 ranked lipids and metabolites (according to mediation proportions) on the associations of chronotype (A and B), chrononutrition (C and D), and uPDI (E and F) with immunophenotypes (for each exposure, only the results of the top 2 immunophenotypes with mediation relationships are shown). Subtitles of the graph represent exposure and immunophenotype in the mediation linkage. The *y* axis represents the ACME of mediation, the *x* axis and the size of the circle represent the proportion of mediation, and the color of the circle represents the direction of mediation (red, D1; blue, D2). Top-ranked lipids or metabolites used to calculate the M-indexes are in bold. (G) A schematic illustration of the construction of the weighted sum of metabolome. The weighted sum of the bidirectional mediation proportions for each lipid or metabolite was calculated to determine its coefficient. These coefficients were then used as weights to calculate the weighted sum of all lipid or metabolite levels, which served as the index. (H) Heatmap showing the correlation between M-indexes and physiological parameters. The color indicates the −log_10_ *P* value times the direction of the association in the general linear model, adjusting for age and gender as confounders. *P < 0.05. (I) uPDI M-index stratified by urban and rural in the Tanzanians cohort [35]. *P* values from paired, 2-tailed Wilcoxon test are shown (*n* = 247 urban residents and 69 rural residents). (J) Chrononutrition M-index stratified by IR and IS in the lipidome cohort [37] (*n* = 35 IR participants and 34 IS participants). *P* values from paired, 2-tailed Wilcoxon test are shown. (K and L) Scatterplot with trend line showing the positive correlation between chrononutrition M-index and BMI in the Rugao cohort [38] (K) or NSPT cohort (L) (3 locations). Global Pearson’s correlation and associated *P* value are shown. The shaded area represents the 95% CI. (M and N) Chrononutrition M-index classified by diabetes in the Rugao cohort [38] (M) or NSPT cohort (N). *P* values from paired, 2-tailed Wilcoxon test are shown. Indexes in the NSPT cohort are adjusted for locations. (O and P) Chrononutrition M-index classified by hypertension in the Rugao cohort [38] (O) or NSPT cohort (P). *P* values from paired, 2-tailed Wilcoxon test are shown. Indexes in the NSPT cohort are adjusted by locations. VAT, visceral adipose tissue; NN, Nanning, China; TZ, Taizhou, China; ZZ, Zhengzhou, China.

In mediation analysis, we found that chronotype effects on immunophenotypes mediated metabolomic shifts, including free fatty acids (FFAs; mean of ACMEs = 0.0066; mean of mediation proportions = 18.25%) and carnitines (mean of ACMEs = 0.0056; mean of mediation proportions = 16.63%; Fig. 5A and B and Table S13). In addition, citrate (mean of ACMEs = 0.00098; mean of mediation proportions = 30.52%) and l-tryptophan (mean of ACMEs = −0.0033; mean of mediation proportions = 16.09%) emerged as key metabolites in the relationships between chronotype and monocyte or mDC subpopulations (Fig. 5B and Table S13). l-Tryptophan, a precursor to 5-hydroxytryptophan and serotonin, plays a crucial role in sleep regulation [31,32]. Lipoproteins from the very-low-density lipoprotein (VLDL) class, which are involved in lipid transportation, showed strong associations with chrononutrition and related immunophenotypes (Fig. 5C and D and Tables S12 and S13). Dietary exposures such as uPDI mediated immunophenotypes by trimethylamine N-oxide (TMAO), glutamic acid, and glutamine (mean of ACMEs = 0.0039; mean of mediation proportions = 15.90%; Fig. 5E and F and Table S13). Glutamine is an essential nutrient for lymphocyte proliferation and cytokine production and is recommended for immunosuppressed individuals [33]. In addition, TMAO plays an important role in cardiovascular and neurological diseases, mediating inflammatory processes, and is considered a promising therapeutic target [34].

We integrated these lipids and metabolites into M-indexes (Fig. S10C to E and Table S14), which constructed analogously to the T-indexes (Fig. 5G). In THPA cohort, the M-indexes were highly correlated with several physiological parameters, including indicators related to obesity, energy metabolism, cardiac health, and glucose homeostasis (*P* < 0.05; Fig. 5H and Table S17). The results showed that unhealthy dietary or sleep habits (higher uPDI and chrononutrition M-index and lower chronotype M-index) were associated with obesity, abnormal energy metabolism, higher blood pressure, and impaired glucose homeostasis. For the uPDI M-index, we observed that it could discriminate between urban and rural populations in Tanzania (2-tailed Wilcoxon test, *P* = 0.002; Fig. 5I), reflecting unhealthier dietary habits, more severe inflammatory status, and higher prevalence of chronic diseases in urban residents [35]. Previous studies have indicated that urban diets are more similar to rural diets during the wet season [35], a trend that was also reflected in the uPDI M-index (Fig. S12C).

Delayed sleep and meal timing has been implicated in an increased risk of metabolic disorders such as obesity and type 2 diabetes [15,16,36]. To understand the role of immunity and metabolism, we calculated the chrononutrition M-index in a lipidome cohort [37] and compared it between participants with insulin resistance (IR) and those who are insulin sensitive (IS). The analysis revealed that the IR group exhibited higher M-indexes, indicating later bedtimes and nocturnal eating habits (2-tailed Wilcoxon test, *P* = 0.00086; Fig. 5J). In addition, M-indexes, including the chronotype, chrononutrition, and uPDI M-indexes, were strongly correlated with BMI. Among these, the chronotype M-index was negatively related to BMI (Fig. S12D and E), whereas the other indexes are positively related to BMI (Fig. 5K and L and Fig. S12F and G). To further assess whether the M-indexes reflect chronic diseases, we evaluated them in the Rugao cohort [38] and the National Survey of Physical Traits (NSPT) cohort, comparing the indexes between individuals with diabetes or hypertension and healthy controls. The results demonstrated that these indexes could effectively distinguish between patients and healthy individuals. Specifically, patients exhibited a higher chrononutrition and uPDI M-index (Fig. 5M to P and Fig. S12H and I) and a lower chronotype M-index (Fig. S12J and K and Table S18) compared to healthy individuals. These findings suggest that the M-indexes are valuable tools for identifying chronic diseases and provide insights into the links among exposures, immunometabolic health, and noncommunicable diseases.

## Discussion

Immunome variation in healthy individuals has attracted considerable research interest, particularly with regard to the factors that drive this variation [4–9,11,39,40]. Our research provided new perspectives on immune variability beyond genetic factors. In this study, we found that sleep and diet are the dominant exposures influencing the human immunome. Sleep mainly influences innate immune cell proportions, while diet affects immune cell surface protein expression. Moreover, we innovatively explored composite exposures that have additional effects on immunophenotypes. Our mediation analysis provided novel molecular insights into exposure-induced immunophenotypes, revealing that sleep interacts with immunophenotypes primarily through transcriptomic changes, whereas diet predominantly influences metabolomic pathways via immunophenotypic alterations. Finally, we developed the T- and M-indexes of exposures, which can reflect the human immune health states and the chronic health conditions.

In addition, the absence of major diseases in our cohort allowed us to better detect the impact of exposure on immunophenotypes, minimizing the confounding effects of diseases. This study not only reported some of the previously known associations between exposure and immunity but also discovered many new signals and revealed more immunological implications affected by exposure. For example, our findings extended the observations of Liu et al. [41] on sleep-deprivation-induced changes in myeloid subsets to more innate immune cell subpopulations. Moreover, we distinguished between the effects of acute and chronic delayed sleep timing on innate immunity, revealing distinct perturbations in IL-1β secretion and in the subpopulations of monocytes and mDCs. The IL-1β secretion stimulated by ATP and LPS indicates the activation of the NLRP3 inflammasome. This process has been reported to be associated with sleep deprivation (SD) and be potentially mediated by substances such as epinephrine, norepinephrine, and reactive oxygen species [42]. In addition, we designed a method to identify lifestyle combinations in this study. One of the interesting combinations we found is chrononutrition. Previous studies have reported its effects on metabolism, methylation, and intestinal immune cells [36,43]. Our results indicate that peripheral immune characteristics further elucidate its biological role, showing that later meal timing is associated with higher levels of inflammatory cytokines.

Another principle contribution of our study is that it focuses on the interactions of immunity with transcription and metabolism, which have been overlooked in previous research on exposure and immunity. Diet acts as a direct source of nutrients and metabolites that directly induce immunophenotypic alterations. That is the reason why there are more D2 mediation linkages among diet, metabolome, and immunome. For poor sleep, both the transcriptome and the immune system are very quick responders. A single night of sleeping loss can lead to significant changes in the proportion of immune cells and immune reprogramming, such as decrease in *GZMB* expression and up-regulation of genes related to inflammatory activation [41]. Apart from genes, the chronotype-induced immune changes are also related to metabolites. This might be partly attributed to organ damage caused by poor sleep and partly to other psychological states or daily behaviors that are associated with sleep. A study in mice demonstrated that prostaglandin D2 efflux across the blood–brain barrier plays an important role in SD induced inflammation [44]. Prolonged SD will further lead to the changes of serum metabolites, such as alanine aminotransferase, aspartate aminotransferase, and urea [44]. The population-based studies further suggested that long-term delayed sleep onset timing is always accompanied by stress [45,46] or poor diet habits [16] and is associated with impaired glycemic health [47]. This requires a more focused research framework for further analysis. However, this study has provided a list of key molecules with their significance in their interrelationships with exposure and immunity.

To further elucidate the health relevance of these exposure-related immune, transcriptional, and metabolic characteristics and to enhance the generalizability of our findings to other populations and scenarios, we designed indexes based on transcriptional and metabolic features, respectively. The comparison between T-index and IHM has proved that these exposure-related immunophenotypes and transcriptional signatures may serve as reliable indicators of immune health. Immune health is a complex concept, and quantifying this state is extremely challenging. The T-indexes can reflect both autoimmune diseases and the responses of the elderly to influenza vaccination and have the potential to become a tool for immune assessment in the future. Moreover, its methodology represents a novel approach for developing such quantitative tools. For the M-indexes, the immune-related metabolomic signatures here showed promise as biomarkers for chronic disease detection. The relationships between M-indexes and chronic health conditions such as BMI, IR, diabetes, and hypertension deepen our understanding of the immune health connotations of exposure-related metabolic signatures. The interrelationship between immune cells and their metabolic environment has been regarded as playing a regulatory role in various cardiovascular diseases, including hypertension [48]. However, future studies should validate these signatures in a prospective cohort to establish their ability to track the occurrence and development of disease independent of therapeutic intervention.

We acknowledge several limitations of this study. First, the number of volunteers with certain adverse behaviors was limited. Second, replication was constrained by the lack of suitable external cohorts with comparable exposure and immunophenotypic data. Third, the observational and cross-sectional nature of our study design limits causal inference between exposures, immunophenotypes, and molecules. Furthermore, exposures may also be the outcome in certain situations, such as when a change in physical status leads to an alteration in behavioral habits. Therefore, further studies require measuring more comprehensive confounders and well-defined experimental validation or longitudinal/intervention-based cohorts to confirm reliability and causality of the associations. In conclusion, our study systematically explores the effects of exposures on the immune system from a multiomics perspective. The T- and M-indexes constructed in this study offer quantitative tools for future health monitoring, demonstrating significant potential for health management applications.

## Materials and Methods

### Population cohort

The 1,001 healthy adult participants of THPA cohort were recruited through the International Human Phenome Project (Shanghai, China) and included 416 males and 585 females aged 20 to 60 years. The inclusion criteria were (a) aged between 20 and 60 years with no plans to move out of Shanghai within 3 years, (b) overall physical health, and (c) understanding and support for the Human Phenome Project. The exclusion criteria were (a) pregnancy or planning pregnancy within 3 years and (b) reported having any of the following major diseases: mental disorders, cardiovascular diseases, respiratory diseases, endocrine diseases, rheumatic and autoimmune diseases, infectious diseases, and any cancer.

This study was approved by the Research Ethics Committee of School of Life Sciences at Fudan University (approval number: BE1828). At recruitment, all 1,001 individuals provided both electronic and written informed consent prior to their involvement in the study.

### Whole blood sampling

Whole blood samples were collected from the 1,001 healthy, fasting donors on heparin tubes, every sampling day from 7:30 AM to 8:30 AM, from 2021 September to 2023 August, in Shanghai, China. The samples were transported to the laboratory at room temperature for processing within 2 h.

### Flow cytometry phenotyping and data handling

Six 11-color flow cytometry panels were developed. Details of staining antibodies and gating strategies can be found in our previous work [49]. Each antibody was selected and titrated as described in the previous work [49], and the staining procedures were optimized for each panel. Samples were acquired using a CytoFLEX LX flow cytometer (Beckman Coulter, Brea, CA, USA). Flow cytometry data were analyzed using CytExpert (Beckman Coulter, version 2.4) and FlowJo software (BD Biosciences, version 10.8). A total of 1,313 flow cytometry measurements were thus analyzed, including 327 cell proportions (including ratios), 302 morphology values, and 684 MFI values (Table S1). Morphology data were quantified by forward scatter (FSC) and side scatter (SSC), where FSC represents cell size and SSC represents cell granularity.

We systematically flagged and eliminated the data with experimental problems, such as abnormal lysis or staining, and then removed the outliers of the immunophenotype whose skewness > 2.15 [if skewness < −2.15, reversed the distribution by normal_rev *< −*max(normal) + 1 − normal] using the method proposed in 2018 [4]. We also corrected for the sampling day batch effect of MFI and morphology immunophenotype, which have relatively large fluctuations with the sampling date, by linear mixed model [Immunophenotype ~ (1|Day of Sampling)], and outliers were removed again. Then, we performed inverse normal transformation for the immunophenotypes whose absolute skewness > 1, while for the other immunophenotypes, we centralized and standardized them using the *scale*() function of R. Missing values were imputed using the *knnImputation*() function of the R package DMwR2 (v0.0.2), and we set the *k* = 5. There was almost no difference in the distribution of data before and after data imputation.

### Whole blood stimulation experiments

Eight immune stimuli (pattern recognition receptor ligands: Pam3CSK4, LPS, flagellin, R848, LPS + ATP; bacteria: Heat-killed *Mycobacterium tuberculosis* (HKMT) and Heat-Killed *Staphylococcus aureus* (HKSA)) were used in the whole blood stimulation experiment. We added 100 μl of whole blood to each well (48-well cell culture plate) and then 400 μl of cell culture medium and immune stimulus (or control [mock]) to each well, mixed gently, and incubated at 37 °C under 5% CO_2_ for 24 h. After stimulation, we centrifuged at 500*g* for 10 min at room temperature. We collected the supernatant to 1.5-ml tube and stored at −80 °C. Details of reagents can be found in our previous work [50].

### Cytokine quantification and data handling

Enzyme-linked immunosorbent assay was performed on the supernatant after 24-h stimulation to quantification cytokines. For LPS (Toll-like receptor 4 [TLR4]), R848 (TLR7/8), and HKSA stimulation, the levels of IL-6 and TNF-α were assessed. For Pam3CSK4 (TLR1/2), flagellin (TLR5), and HKMT stimulation, only IL-6 concentrations were assessed. For LPS + ATP (NLRP3) stimulation, we assessed the concentration of IL-1β. Details of the experimental procedure can be found in our previous work [50]. Besides, plasma levels of 10 inflammatory factors, including IL-2, IL-4, IL-6, IL-8, IL-10, IL-12p70, IL-13, IL-1β, IFN-γ, and TNF-α were quantified using the V-PLEX assay on the MESO QuickPlex SQ 120 (Meso Scale Discovery [MSD], Rockville, USA). The unit of cytokines is picograms per milliliter. We found that the distribution of cytokines was also skewed to the right, so we performed the same data preprocessing strategy as described in the “Flow cytometry phenotyping and data handling” section. Only the poststimulation cytokines were corrected for the sampling day batch effect using a linear mixed model [Immunophenotype (Cytokines after stimulation) ~ (1|Day of Sampling)].

### Inclusion and imputation of candidate exposures

A total of 183 exposures obtained by self-reported questionnaire were selected for this study, and they were classified into 12 categories: alcohol use, antibiotic use, characteristics of residences, dietary behavior, dietary intake, mental health, physical activity, sleep, sociodemographics, tobacco smoke factors, ultraviolet light exposures and vitamins, minerals, and other supplement use (Table S2). The classification criteria for these exposures were based on the Personalized Environmental and Genetics Study (PEGS) [51] and the Human Early Life Exposome (HELIX) [52] projects. All these exposures were quality controlled and recoded one by one. If the answer to the questionnaire was irrational, it was recoded as NA. In addition, the included exposures were ensured to have more than 30 observations. According to the time of start and end, we calculated the number of years of smoking and drinking. The metabolic equivalent in our cohort was calculated using the formula reported previously [53].

For dietary exposures, energy intakes exceeding 800 to 4,200 kcal/d for men and 500 to 3,500 kcal/d for women were considered extreme values and excluded. To be consistent with previous Chinese cohort studies [54], a total of 16 food groups (milk and dairy products, sugar, meat, fresh fruits, fresh vegetables, fish and aquatic products, legumes, whole grains, refined grains, tea, garlic, nuts, eggs, preserved vegetables, vegetable oils, and animal fat) [54] were used to assess the plant-based dietary patterns (PDI, healthful PDI, and uPDI) in the current study. The intake frequency of different food groups was calculated by summing the intake frequency of all the related foods in that group, and the food items constituting the 16 food groups were referred to previous studies [55–57]. Next, the intake frequency of each food group was equally divided into 3 groups, with the high, medium, and low groups being assigned 5, 3, or 1 (for binary answers, assigned 5 or 1). These 16 food groups were classified as healthful plant foods, unhealthful plant foods, and animal foods, and the other scoring strategies were based on the study reported by Chen et al. [54]. In addition, all the continuous diet variables (as dietary intake) were adjusted for total energy intake after centralization and standardization.

For the continuous variables, we further centralized and standardized them using the *scale*() function of R. Missing values were imputed using the *knnImputation*() function of the R package DMwR2 (v0.0.2), and we set the *k* = 5. There was almost no difference in the distribution of data before and after data imputation.

### RNA sequencing of whole blood samples

The transcriptome was evaluated using RNA sequencing (RNA-seq) of bulk whole blood, which was stored in liquid nitrogen. Briefly, total RNA was extracted using the miRNeasy Mini Kit (QIAGEN), quantified with a Qubit Flex fluorometer (Invitrogen), and assessed for quality using a 4,200 TapeStation System (Agilent Technologies). Ribosomal and globin RNAs were then depleted using probes (Vazyme, Nanjing, Jiangsu Province, China), and libraries were constructed with the VAHTS Universal V8 RNA-seq Kit (Vazyme, China). Library quantification and size distribution were evaluated using a Qubit Flex fluorometer and Bioptic Qsep100. Finally, the libraries were sequenced on the Illumina NovaSeq 6000 platform with 150-bp paired-end reads. The details of this procedure have been described by Yu et al. [58].

After sequencing, fastp (v0.19.6) was used to remove adapters from the raw reads, followed by HISAT (v2.1), SAMtools (v1.3.1), StringTie (v1.3.4), and Ballgown (v2.14.1) for alignment and gene quantification using the GRCh38 human genome and Ensembl gene models as references [59]. Then, gene counts were calculated through Ballgown, and log_2_-transformed expression profiles were generated using edgeR (v3.24.3) after fragments per kilobase of transcript per million mapped reads (FPKM) normalization. Genes with low expression in more than 30% of the samples were eliminated. In addition, we corrected for the batch effect using the *ComBat*() function of the sva (v3.46.0) R package and removed the outliers that were outside the 3-fold standard deviation of the values. After these steps, we had 991 samples with expression data from 20,562 genes.

### Plasma metabolome

Plasma samples from study participants were stored at −80 °C. During extraction, plasma samples were thawed on ice, vortexed, and spun down. We conducted the combined nuclear magnetic resonance and high-performance liquid chromatography-tandem mass spectrometry methods to quantify the metabolome (lipids and metabolites). Metabolites included elementome [60], amino metabolites [61], acyl-carnitines [62], lipoprotein subfraction and components [63], and sterol metabolites and bile acids [64]. All lipids and metabolites were identified using in-house library reference standards based on their endogenous retention index [65], mass-to-charge ratio, and mass spectrometry spectral data. We checked the batch effect of the data, eliminated metabolites that were not detected in more than 80% of the samples, and performed the log transformation by log(*x* + 1) in R. We then removed the outliers that were outside the 3-fold standard deviation of the values. A total of 2,020 annotated metabolites and lipids from 995 samples were retained for downstream analysis.

### Health-related data collection

Five categories of health-related data were included in this study: glucose homeostasis related data, blood pressure data (cardiac data), energy metabolic data, and anthropometric data. The biochemical data, plasma levels of 8 metabolic factors, including active glucagon-like peptide-1 (active_GLP-1), active human glucose-dependent insulinotropic polypeptide (human_GIP-active), C-peptide, fibroblast growth factor 21 (FGF-21), FGF-23, glucagon, insulin, and leptin, were quantified using the U-PLEX assay on the MESO QuickPlex SQ 120 (MSD, Rockville, USA). Blood pressure data included systolic blood pressure and diastolic blood pressure. Waist circumference (WC) and waist-to-hip ratio (WHR) were assessed using the 3-dimensional body scanning system, while detailed body compositions (visceral adipose tissue volume and visceral adipose tissue mass) were assessed using dual-energy x-ray absorptiometry. Energy metabolic data included resting metabolic rate, energy expenditure, and basal metabolic rate. We transformed them using inverse normal transformation. All of these health-related data are listed in Table S16.

### Genotype information

DNA were extracted from blood samples using the DNeasy Blood & Tissue Kit (QIAGEN, Germany) and measured by the Qubit DNA Assay Kit (Life Technologies, USA). DNA libraries were generated using MGIEasy Universal DNA Library Prep Kit (MGI, China) following the manufacturer’s recommendations. Then, the libraries constructed above were sequenced by DNBSEQ-T7 platform (MGI, China), and 150-bp paired-end reads were generated for further quality control and data analysis. The first 20 genetic PCs of the PC analysis (PCA) on the individual genotypes of 993 volunteers in THPA cohort were evaluated and used for further analyses.

### Distance-matrix-based immunome variance estimation

The contributions of different factors, including exposures and covariates, to interindividual immunome variations were estimated by permutational multivariate analysis of variance (PERMANOVA) using distance matrices. First, each exposure was used to estimate interindividual immunome variations using the *adonis*() function from the R package vegan (v2.6-10; https://cran.r-project.org/web/packages/vegan/index.html) with 1,000 permutations. Only exposures that could estimate interindividual immunome variation with a permutation FDR of <0.05 were retained. For exposures showing an association (Spearman’s correlation coefficient > 0.4), only the exposure with the largest contribution to immunome variation (on the basis of *R*^2^ values) was retained. All representative exposures were further included in a multivariate PERMANOVA to estimate the combined contribution to interindividual immunome variation. When comparing the contributions of different exposures to variation in the immunome, the effects of age, gender, and season on immunophenotypes were adjusted by multivariate linear regression prior to PERMANOVA.

### Identification of composite exposures

We computed the Spearman’s correlation matrix of exposures and set the coefficient of correlations that Bonferroni-adjusted *P* ≥ 0.05 or |*r*| ≤ 0.2 (median of absolute *r*) to 0 to remove the noise generated by insignificant correlations. Then, we performed unsupervised ward clustering of the correlation based on Euclidean distances using the pheatmap package (v1.0.12) in R and extracted shared information from exposures in different clusters with high correlations by a partial least-squares path model using the R package plspm (v0.5.1) [66]. We tested the clustering results of different thresholds of |*r*| (0, 0.1, 0.2, and 0.3) and chose 0.2 because it neither generated excessive noise in the modules nor resulted in overly small modules that would cause information loss. To determine the number of composite exposures, we sequentially increased the number of clusters until there was no significant correlation between the constructed composite exposures. We found that setting the number of clusters to 13 allowed us to construct 12 composite exposures, among which no significant associations were found. Details on the compositions of these composite exposures can be found in Table S8.

To further ensure that the data imputation does not affect the construction results of the composite exposures, we also conducted an unsupervised clustering analysis using the data before imputation. The results showed that the clusters of exposures obtained (Fig. S8B) were not significantly different from those obtained after imputation (Fig. S8A).

### Association, resampling, and stratified analysis

All 183 exposures were tested for associations with the 4 categories of immunophenotypes separately through *T*-test testing of coefficient estimates and LRTs, using age, gender, and season as covariates: the full model is lm(immunophenotype ~ exposure + age + gender + season), and the null model is lm(immunophenotype ~ age + gender + season), followed by Benjamini–Hochberg multiple testing correction. The FDRs of the coefficients were generally below the FDRs of the LRTs, so we used the FDR of the coefficients as the final standard, and if it was below 0.05, it was considered statistically significant (this procedure was repeated for 12 composite exposures). We also applied the stratified analysis by gender and age, using the same method as above (gender, male versus female; age, less than 40 years old versus greater than or equal to 40 years old). We presented the results of the stratified analysis of 59 significant associations (Fig. 2C to F and Table S6) and the significant associations in different subgroups based on FDR (FDR < 0.05; Table S7).

To assess the robustness of the effect of exposures on immunophenotypes, we randomly selected 90% and 80% of all the samples and performed statistical tests on each association. We repeated this 100 times and recorded the number of *P* values below 0.05 and the mean of the *P* values and coefficients. To demonstrate that the results are not an artifact of the data imputation method, we repeat the analysis on a subset of samples with complete data. All the associations remained significant; details are provided in Table S3.

### Stratified analysis of MEQ score

For the stratified analysis according to chronotype (quantified by MEQ score), we divided the population according to MEQ scores. Individuals with MEQ score ≤ 41 are of evening chronotype, individuals with MEQ score ≥ 59 are of morning chronotype, and the others are of middle chronotype. In each group, we performed linear regression: lm(significant immunophenotype ~ nocturnal sleep restriction + age + gender + season). To further eliminate the influence of age on sleep habits, we corrected the sleep factors based on age and then repeated the above regression process.

### Bidirectional mediation analysis

We adjusted the immunome, transcriptome, and metabolome for the basic covariates (age, gender, and season) and selected genes, lipids, and metabolites associated with both exposures (Spearman’s correlation, FDR < 0.05) and immunophenotypes (Spearman’s correlation, FDR < 0.05). Immunophenotypes and exposures here are significant in exposure–immunophenotype association analysis (see the “Association, resampling, and stratified analysis” section for details). Next, we carried out bidirectional mediation analysis with interactions (*y* = *x* + *m* + *x* × *m*, where *y* is the outcome, *x* is the exposure, and *m* represents the mediator) between mediator and outcome taken into account using the *mediate* function from mediation (v4.5.0) [67] with 1,000 bootstrap sampling times to infer the mediation effect of transcripts, metabolites on immunophenotype (D1 mediation: FDR [D1] < 0.05, mediation proportion [D1] > mediation proportion [D2]), and the mediation effect of immunophenotypes on transcripts or metabolites (D2 mediation: FDR [D2] < 0.05, mediation proportion [D2] > mediation proportion [D1]). The FDR was calculated using the Benjamini–Hochberg procedure. Specifically, 2 linear models were fitted as follows:

For the D1 mediation:

$${\text{Omics}}_{i}=\alpha 1+\beta 1{\text{Expo}}_{i}+\delta 1{\text{Omics}}_{i}\times {\text{Expo}}_{i}+\varepsilon_{i}1$$ (1)

$${\text{Immuno}}_{i}=\alpha 2+\beta 2{\text{Omics}}_{i}+\gamma 1{\text{Expo}}_{i}+\delta 2{\text{Omics}}_{i}\times {\text{Expo}}_{i}+\varepsilon_{i}2$$ (2)

For the D2 mediation:

$${\text{Immuno}}_{i}=\alpha 1+\beta 1{\text{Expo}}_{i}+\delta 1{\text{Omics}}_{i}\times {\text{Expo}}_{i}+\varepsilon_{i}1$$ (3)

$${\text{Omics}}_{i}=\alpha 2+\beta 2{\text{Immuno}}_{i}+\gamma 1{\text{Expo}}_{i}+\delta 2{\text{Omics}}_{i}\times {\text{Expo}}_{i}+\varepsilon_{i}2$$ (4)

where *Omics_i_* represents each omics feature (transcripts and metabolites), *Expo_i_* represents each exposure, and *Immuno_i_* represents immunophenotypes. After fitting these 2 modules, the product of the 2 coefficients β1β2 was interpreted as an estimate of the ACME, and the coefficient γ1 was interpreted as an estimate of the average direct effect (ADE), and the γ1+β1β2 was interpreted as an estimate of the total effect. The proportion mediation proportion of the effect of the exposure on the outcome that was mediated by the mediator was calculated as follows:

$$\text{Mediation Proportion}=\frac{\text{ACME}}{\;\text{Total Effect}}=\frac{\text{ACME}}{\;\mathrm{ADE}+\text{ACME}}=\frac{\beta 1\beta 2}{\;\gamma 1+\beta 1\beta 2}$$ (5)

### Calculation of T- or M-indexes

To quantify the mediation effect of exposure, molecules and immunophenotypes, we obtained the sum of the D1/D2 mediation proportion times the effect direction of each transcript or metabolite and used this as the weight to calculate the following 5 T- or M-indexes: nocturnal sleep restriction T-index, chronotype T-index, chronotype M-index, uPDI M-index, and chrononutrition M-index. For a specific exposure, the calculation formula is as follows:

$${\text{PropSum}}_{k}=\sum_{i}^{j}m_{k,i}{\mathrm{MP}}_{k,i}$$ (6)

mk,i=−1,ACMEk,i<01,ACMEk,i>0 (7)

$$T-\left(\text{or}\;M-\right)\;\text{index}=\sum_{k}^{l}{\text{PropSum}}_{k}{\text{Omics}}_{k}$$ (8)

where ${\mathrm{MP}}_{k,i}$ represents the mediation proportion of *immunophenotype_i_* and ${\text{Omics}}_{k}$ (*Gene_k_*, *lipid_k_* or *metabolite_k_*); $m_{k,i}$ represents the ACME direction of *immunophenotype_i_* and ${\text{Omics}}_{k}$ (*Gene_k_*, *lipid_k_*, or *metabolite_k_*); *immunophenotype_i_* represents the immunophenotype that has mediation effect with ${\text{Omics}}_{k}$ and a certain exposure (nocturnal sleep restriction, chronotype, uPDI, and chrononutrition); ${\text{Omics}}_{k}$ represents the normalized level of *Gene_k_*, *lipid_k_*, or *metabolite_k_*, which is involved in the mediation linkages of a certain exposure (nocturnal sleep restriction, chronotype, uPDI, and chrononutrition). The *PropSum* (representing the weight or coefficient) of the molecule was shown in Table S14. The transcriptome and metabolome were imputed by mean values using the *impute*() function of the R package e1071 (v1.7-16).

Given the high correlation between whole blood RNA-seq data and immunophenotypic data, we further defined each gene in the mediation linkages as an immune-cell-specific gene (Table S14) based on the CellMarker 2.0 database (http://117.50.127.228/CellMarker/).

T-indexes were tested for association with IHM transcriptional surrogate signature score [23] by linear regression, using age and gender as covariates: lm(IHM ~ index + age + gender). The IHM transcriptional surrogate signature score was calculated in THPA cohort using tools at https://panmonogenic.yale.edu/ [23]. Results of this test are shown in Table S18.

M-indexes were tested for association with the health-related data by linear regression, using age and gender as covariates: lm(health-related data ~ index + age + gender), followed by Benjamini–Hochberg multiple testing correction, and were considered statistically significant if the adjusted *P* value was below 0.05. Results are shown in Table S17.

### Validation of T- or M-indexes in the external cohorts

The T- or M-indexes in the 8 external cohorts were calculated to assess whether the indexes could well reflect immune health and chronic diseases. The detailed information of these 8 external cohorts can be found in Fig. S11 and Table S15. The NSPT cohort included 3,557 Chinese individuals recruited as volunteers from 3 regional districts in China: Zhengzhou, Taizhou, and Nanning. Serum metabolites of the NSPT cohort were examined using previously reported methods [68]. In this study, 351 metabolomic parameters were obtained form 3,037 human serum samples.

In the SLE cohort [25], RA cohort [26], vaccination cohort [29], and COVID-19 cohort [30], we calculated chronotype T-index and nocturnal sleep restriction T-index using overlapping genes. We performed Pearson’s correlation to test association between these T-indexes and disease activity score in 28 joints (DAS28) or CRP levels in RA. For the other conditions, we performed a 2-tailed Wilcoxon test. Furthermore, logistic regression or linear regression was used to correct for the effect of gender, age, BMI, race, or treatment on the outcomes. In the SLE cohort, we fitted mixed-effects models: glm(healthy or SLE ~ index + age + gender + race + treatment + 1|subject) and lm[log(SLEDAI + 1) ~ index + age + gender + race + treatment + lymphocyte count + neutrophil count + 1|subject]; in the RA cohort, we performed linear regression: lm[DAS28 (or CRP) ~ index + age + gender + BMI + ethnicity + treatment]; in the COVID-19 cohort, we performed multinomial logistic regression: multinom(healthy, mild, or severe ~ index + age + gender) and logistic regression: glm(survivor or fatality ~ index + age + gender). SLE disease activity index (SLEDAI) score reflects disease activity of SLE. The results are shown in Table S18.

In the Tanzanians cohort [35], lipidome cohort [37], Rugao cohort [38], and NSPT cohort, we calculated chronotype M-index, chrononutrition M-index, and uPDI M-index using overlapping lipids and metabolites. We used Pearson’s correlation to test the association between these M-indexes and BMI. For the other conditions, we conducted a 2-tailed Wilcoxon test. As above, logistic regression or linear regression was further used to correct for the effect of gender, age, or BMI on the outcomes. In the lipidome cohort, we performed logistic regression: glm(IR or IS ~ index + age + gender + BMI); in the Rugao and NSPT cohorts, we performed linear regression: lm(BMI ~ index + age + gender) and logistic regression: glm(healthy or disease ~ index + age + gender + BMI + treatment). The indexes of the NSPT cohorts were adjusted for locations. In the Tanzanians cohort, we adjusted the uPDI M-index for age and gender and performed a 2-tailed Wilcoxon test. The results are shown in Table S18.

To directly test the influence of medication, we compared the levels of M-index between patient subgroups (patients with or without medications) in Rugao and NSPT cohorts. We found no significant difference in M-indexes between these groups (*P* ≥ 0.05; Fig. S13), even after the adjustment of age, sex, and BMI. Furthermore, we compared M-indexes across people with different number of diseases (0, 1, and 2; diseases include diabetes and hypertension) and found changes in the gradient (Fig. S14).

### Statistical analysis

All statistical analyses were performed using R (v4.4.1 and v4.2.3), and RStudio (v2023.03.0+386 and v2024.04.2). Spearman’s correlation coefficient was used for pairwise correlation comparisons of exposures, while Pearson’s correlation coefficient was used for other correlation analyses (as the intracorrelation analysis of the immunome). FDRs or Bonferroni-adjusted *P* value below 0.05 were considered statistically significant. IQR of immunophenotypes was calculated using *IQR*() in R.

To test the association of immunophenotypes with age, we corrected for the effects of gender and season using a linear model. Spearman’s correlation analysis was used to determine the significance and direction of the correlations between age and immunophenotypes. To test whether gender had any effect on the immunophenotypes, we used a linear model to correct for the effect of age and season. A paired *t* test was carried out between the gender-stratified samples. To investigate the effect of season (12 months) on the immunophenotypes measured, we used a linear model to correct for the effect of age and gender, normalized the immunophenotypes using *x* − min(*x*)/max(*x*) − min(*x*) and performed the *circa_single*() function of the R package circacompare (v0.2.0). Significance of the above tests was all reported after controlling for multiple testing (FDR < 0.05).

We performed the sensitivity analysis for all significant exposure and immunophenotype pairs. We evaluated the effect of exposure on immunophenotypes when more covariates (BMI, COVID-19 vaccination, or COVID-19) were added: compare lm(immunophenotype ~ exposure + age + gender + season + BMI) with lm(immunophenotype ~ age + gender + season + BMI); compare lm(immunophenotype ~ exposure + age + gender + season + COVID-19 vaccination + COVID-19) with lm(immunophenotype ~ age + gender + season + COVID-19 vaccination + COVID-19). In addition, we formally checked how the genetic background of the donors could affect immunophenotypes by performing association tests between the first 20 genetic PCs of the PCA on the individual genotypes and each of the significant immunophenotype. We evaluated the effect of exposure on immunophenotype, adding the first 20 genetic PCs associated with this immunophenotype as covariate: compare lm(immunophenotype ~ exposure + age + gender + season + the genetic PCs associated with this immunophenotype) with lm(immunophenotype ~ age + gender + season + the genetic PCs associated with this immunophenotype).

## Ethical Approval

This study was approved by the Research Ethics Committee of School of Life Sciences at Fudan University (approval number: BE1828). At recruitment, all 1,001 individuals provided both electronic and written informed consent prior to their involvement in the study.

## Data Availability

Exposure data, health-related data, and immunophenotypic data have also been deposited to PhenoBank repository (https://www.phenobank.org.cn, a data sharing and collaborative research platform for human phenome data) and HPCDE (Human Phenome Common Data Element) Portal (https://www.biosino.org/hpcde) gradually. Transcriptomic data and genomic data are deposited in NGDC (National Genomics Data Center; https://ngdc.cncb.ac.cn/) with accession number: PRJCA026248 (RNA-seq data, OMIX006455, https://ngdc.cncb.ac.cn/omix/preview/HrkIPdsr) and PRJCA026249 (whole-genome sequencing data, GVM000761, https://bigd.big.ac.cn/gvm/getProjectDetail?Project=GVM000761). The sequencing data are available under controlled access due to data privacy laws related to volunteer consent for data sharing, and the data should be used for research purposes only. All the other data are available after submitting the application and obtaining approval. The metabolomic data and all the related association results in this paper are available upon request. The detail information of 8 external datasets can be found in Table S15. Source data of main figures are provided with this paper (Data S1 to S5). Software code and source data for reproducing our analyses are available at https://github.com/zzzyyr123/exposure-immune-omics-map. Any additonal information required to reanalyze the data reported in this paper is available upon request.

## Appendix A

### Members of Human Phenome Project Scientific Consortium

Jun Chang, Wei Chen, Chen Ding, Li Jin, Tiemin Liu, Jingchun Luo, Qiang Luo, Yuan Qi, Feng Qian, Leming Shi, Dean Ta, Huiru Tang, Weishuo Tao, Mei Tian, Jiucun Wang, Sijia Wang, Shuhua Xu, Zhiqiang Yan, Zhong Yang, Guoqing Zhang, Menghan Zhang, Yan Zheng, Lei Zhu.

#### Affiliations

State Key Laboratory of Genetics and Development of Complex Phenotypes, Human Phenome Institute, Fudan University, Shanghai 200433, China (J.C., W.C., C.D., L.J., L.T., J.L., L.Q., F.Q., L.S., D.T., H.T., W.T., M.T., J.W., S.X., Z. Yan, Z. Yang, M.Z., Y.Z., L.Z.); Artificial Intelligence Innovation and Incubation Institute, Fudan University, Shanghai 201203, China (Y.Q.); Shanghai Institute of Nutrition and Health, University of Chinese Academy of Sciences, Chinese Academy of Sciences, Shanghai 200031, China (S.W.); Bio-Med Big Data Center, Chinese Academy of Sciences Key Laboratory of Computational Biology, Shanghai Institute of Nutrition and Health, University of the Chinese Academy of Sciences, Chinese Academy of Sciences, Shanghai 200031, China (G.Z.).

### Members of Human Phenome Project Technological Consortium

Ran An, Wenyan Chai, Chen Chen, Longlong Chen, Qinsheng Chen, Weixin Chen, Xingdong Chen, Qiming Dai, Wenxue Ding, Yan Fang, Jinwen Feng, Lei Feng, Shujing Fu, Jian Gao, Jieyu Ge, Jiani Gu, Qingxia Huang, Wanwan Hou, Liguo Jia, Nan Jiang, Wan Jiang, Mengmeng Kong, Xuan Lan, Anxin Li, Hao Li, Jinxi Li, Jing Li, Ting Li, Wenyu Li, Yao Li, Yingcen Li, Huiqin Lin, Quanzhong Liu, Ting Liu, Yangyang Liu, Jingjing Luo, Lan Luo, Quanne Niu, Yujie Pan, Xiaolan Qian, Xiaoqing Qiao, Zhaoyu Qin, Zhiwei Rong, Bing Shen, Shengli Shen, Shiwei Shen, Biru Shi, Jing Sun, Zhonghan Sun, Liangyan Tang, Mengting Wan, Chengyan Wang, He Wang, Jialin Wang, Miaomiao Wang, Qi Wang, Qiying Wang, Rui Wang, Yun Wang, Yunzhi Wang, Lingge Wu, Xin Xia, Binxin Xu, Yongwu Xu, Zhipeng Xu, Liping Xue, Hui Xie, Jun Yan, Cuiwei Yang, Ying Yu, Hui Yuan, Chao Zhang, Manfei Zhang, Wenjing Zhang, Yining Zhao, Yuanting Zheng, Chen Zhu, Lingli Zhu.

#### Affiliation

State Key Laboratory of Genetics and Development of Complex Phenotypes, School of Life Sciences, Human Phenome Institute, Fudan University, Shanghai 200433, China.
