# Supplementary material for: The Influence of Sleep and Diet on Human Peripheral Immunity and Chronic Health Conditions
Source: Research (Wash D C). 2026 Feb 19;9:1081. doi: 10.34133/research.1081 (PMC12943795; doi:10.34133/research.1081)
Supplement: Supplementary 1 — Figs. S1 to S14 Tables S1 to S18 Data S1 to S5 [file research.1081.f1.zip › Fig S1.pdf]

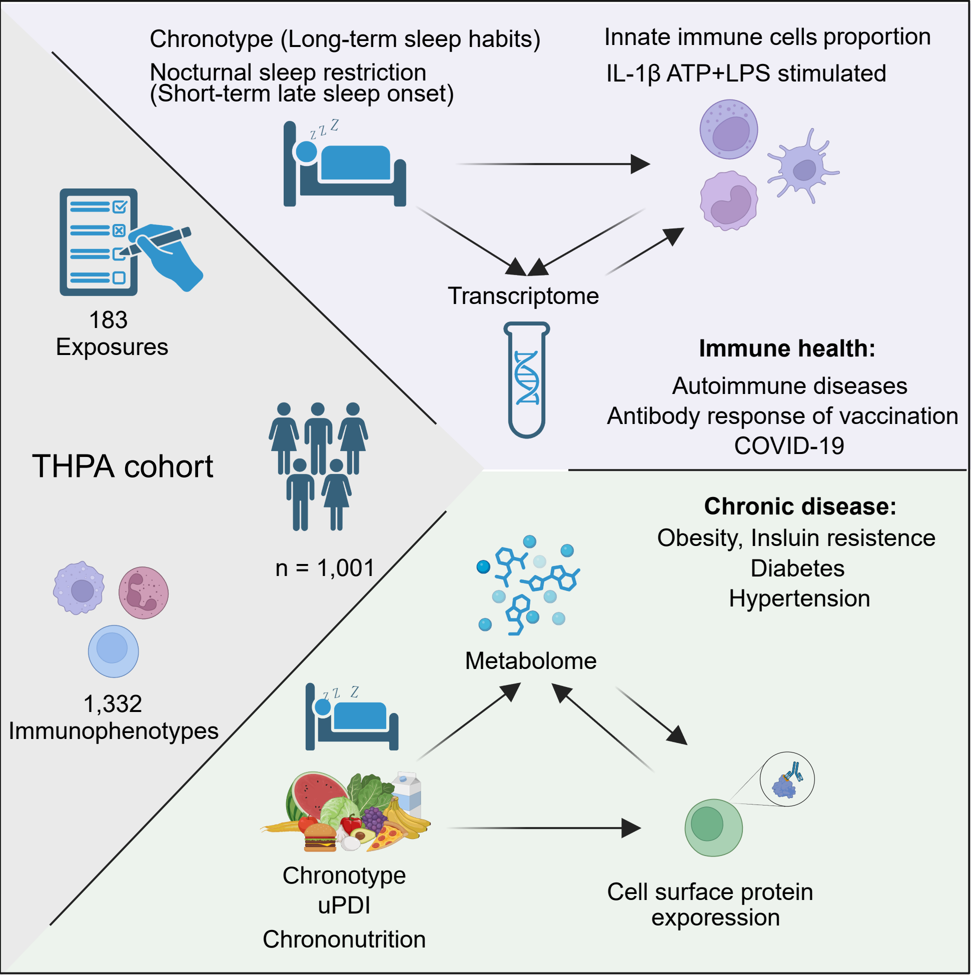
Chronotype (Long-term sleep habits)
Nocturnal sleep restriction
(Short-term late sleep onset)
Innate immune cells proportion
IL-1β ATP+LPS stimulated
183
Exposures
Transcriptome
Immune health:
Autoimmune diseases
Antibody response of vaccination
COVID-19
THPA cohort
n = 1,001
Chronic disease:
Obesity, Insluin resistence
Diabetes
Hypertension
1,332
Immunophenotypes
Metabolome
Chronotype
uPDI
Chrononutrition
Cell surface protein
exporession
