# Supplementary material for: The Influence of Sleep and Diet on Human Peripheral Immunity and Chronic Health Conditions
Source: Research (Wash D C). 2026 Feb 19;9:1081. doi: 10.34133/research.1081 (PMC12943795; doi:10.34133/research.1081)
Supplement: Supplementary 1 — Figs. S1 to S14 Tables S1 to S18 Data S1 to S5 [file research.1081.f1.zip › Fig S11.pdf]

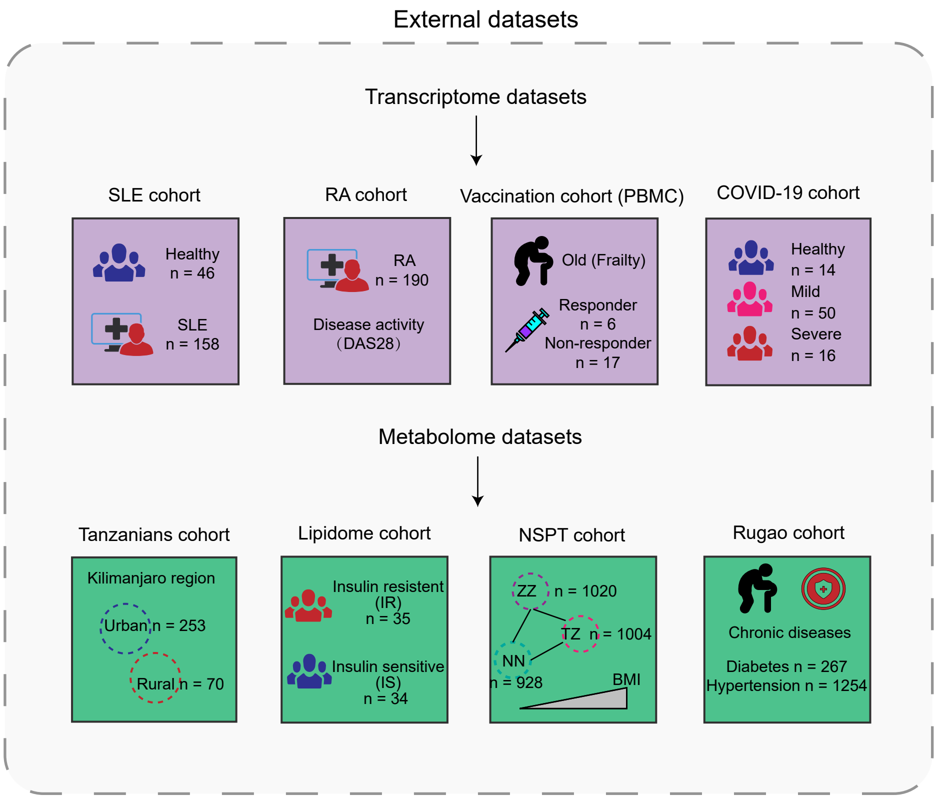
External datasets
Transcriptome datasets
SLE cohort
Healthy
n = 46
SLE
n = 158
RA cohort
RA
n = 190
Disease activity
（DAS28）
Vaccination cohort (PBMC)
Old (Frailty)
Responder
n = 6
Non-responder
n = 17
COVID-19 cohort
Healthy
n = 14
Mild
n = 50
Severe
n = 16
Metabolome datasets
Tanzanians cohort
Kilimanjaro region
Urban n = 253
Rural n = 70
Lipidome cohort
Insulin resistent
(IR)
n = 35
Insulin sensitive
(IS)
n = 34
NSPT cohort
ZZ n = 1020
TZ n = 1004
NN
n = 928
BMI
Rugao cohort
Chronic diseases
Diabetes n = 267
Hypertension n = 1254
