# Supplementary material for: The Influence of Sleep and Diet on Human Peripheral Immunity and Chronic Health Conditions
Source: Research (Wash D C). 2026 Feb 19;9:1081. doi: 10.34133/research.1081 (PMC12943795; doi:10.34133/research.1081)
Supplement: Supplementary 1 — Figs. S1 to S14 Tables S1 to S18 Data S1 to S5 [file research.1081.f1.zip › Fig S5.pdf]

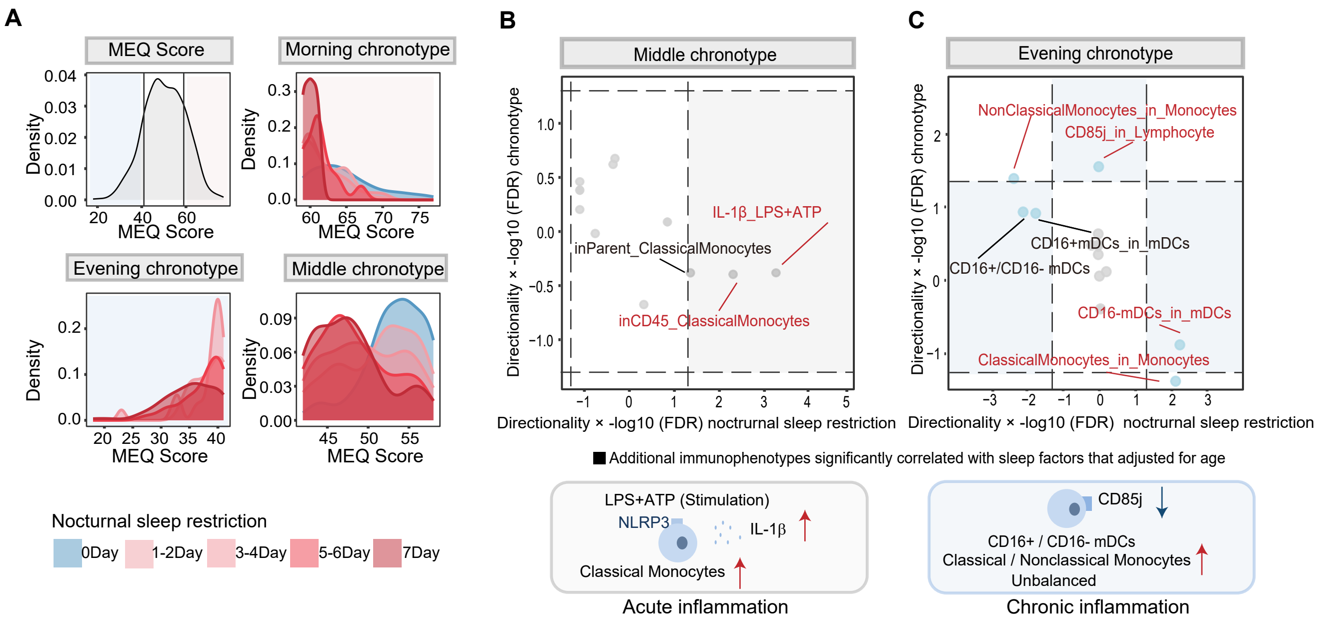

A
MEQ Score
Morning chronotype
Evening chronotype
Middle chronotype
Density
MEQ Score
Nocturnal sleep restriction
0Day
1-2Day
3-4Day
5-6Day
7Day
B
Middle chronotype
Directionality × -log10 (FDR) chronotype
Directionality × -log10 (FDR) noctrunal sleep restriction
IL-1β_LPS+ATP
inParent_ClassicalMonocytes
inCD45_ClassicalMonocytes
C
Evening chronotype
NonClassicalMonocytes_in_Monocytes
CD85j_in_Lymphocyte
CD16+mDCs_in_mDCs
CD16+/CD16- mDCs
CD16-mDCs_in_mDCs
ClassicalMonocytes_in_Monocytes
Directionality × -log10 (FDR) noctrunal sleep restriction
Additional immunophenotypes significantly correlated with sleep factors that adjusted for age
LPS+ATP (Stimulation)
NLRP3
IL-1β
Classical Monocytes
Acute inflammation
CD85j
CD16+ / CD16- mDCs
Classical / Nonclassical Monocytes
Unbalanced
Chronic inflammation
