# Supplementary material for: The Influence of Sleep and Diet on Human Peripheral Immunity and Chronic Health Conditions
Source: Research (Wash D C). 2026 Feb 19;9:1081. doi: 10.34133/research.1081 (PMC12943795; doi:10.34133/research.1081)
Supplement: Supplementary 1 — Figs. S1 to S14 Tables S1 to S18 Data S1 to S5 [file research.1081.f1.zip › Fig S8.pdf]

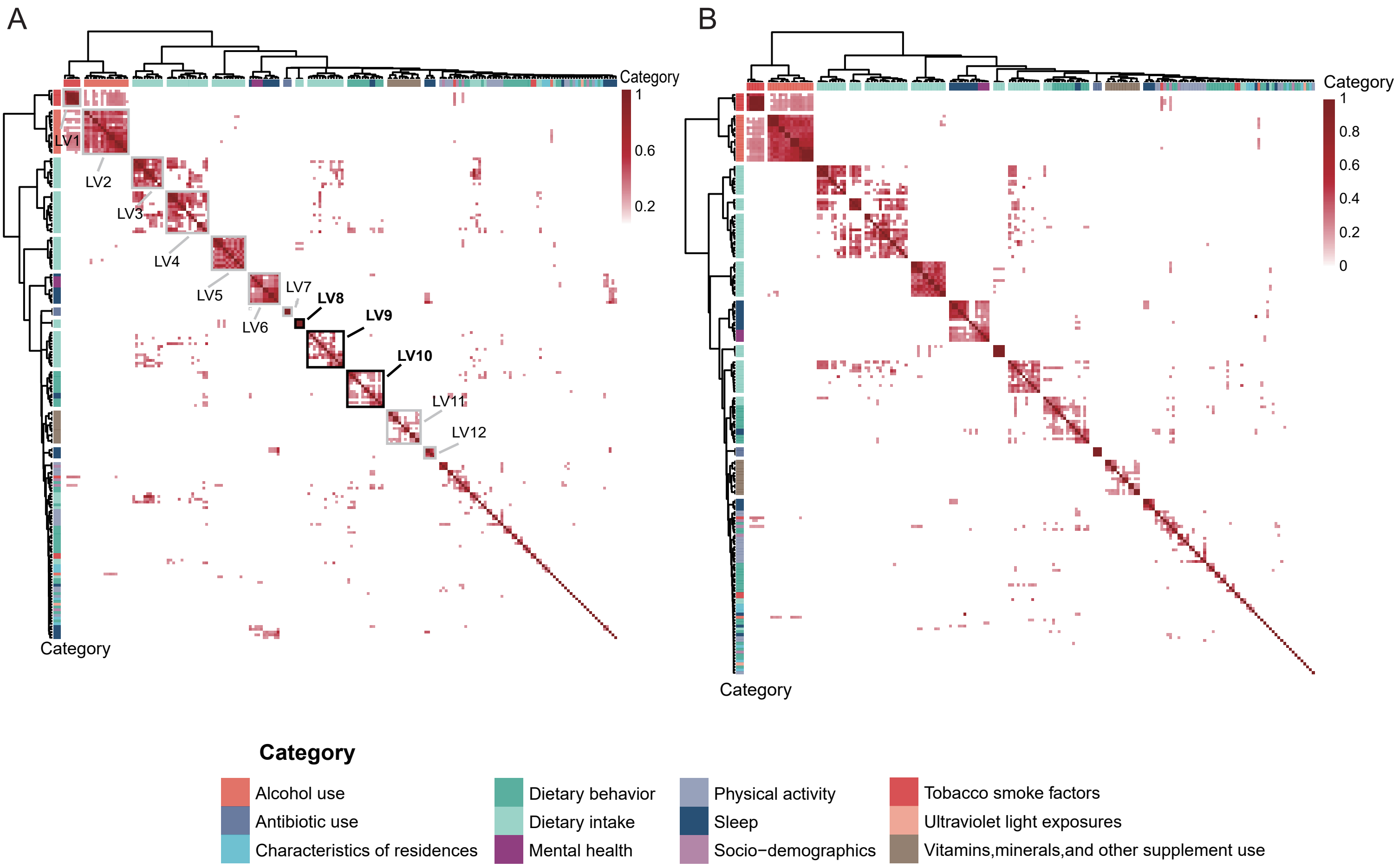
A
B
Category
1
0.6
0.2
0.8
0.4
0
LV1
LV2
LV3
LV4
LV5
LV6
LV7
LV8
LV9
LV10
LV11
LV12
Alcohol use
Antibiotic use
Characteristics of residences
Dietary behavior
Dietary intake
Mental health
Physical activity
Sleep
Socio−demographics
Tobacco smoke factors
Ultraviolet light exposures
Vitamins,minerals,and other supplement use
