# Supplementary material for: The Influence of Sleep and Diet on Human Peripheral Immunity and Chronic Health Conditions
Source: Research (Wash D C). 2026 Feb 19;9:1081. doi: 10.34133/research.1081 (PMC12943795; doi:10.34133/research.1081)
Supplement: Supplementary 1 — Figs. S1 to S14 Tables S1 to S18 Data S1 to S5 [file research.1081.f1.zip › Research-Supplemental Information-revision.docx]

Supplemental Information

The Influence of Sleep and Diet on Human Peripheral Immunity and Chronic Health Conditions

Yiran Zhao^1,2※^, Wenran Li^2※^, Bingjie Li^2※^, Siyu Zhou^1^, Xianlei Zhao^1^, Qi Wang^1^, Yingyu Cheng^2^, Yali Luo^1^, Jingxuan Han^1^, Xuling Han^1^, Helian Li^1^, Jian Gao^1^, Jialin Zhao^1^, Zhonghan Sun^1^, Mengmeng Kong^1^, Xiaofeng Zhou^1^, Ying Yu^1^, Wanwan Hou^1^, Qinsheng Chen^1^, Jingxian Zhang^1^, Xiaofeng Wang^1^, Jingchun Luo^1^, Li Jin^3^, Leming Shi^5^, Yan Zheng^3^, Human Phenome Project Technological Consortium, Human Phenome Project Scientific Consortium, Huiru Tang^3^*, Sijia Wang^2^*, Feng Qian^1, 3, 4, 5, 6, 7^*

※These authors contributed equally to this work.

*Corresponding author. Email: [huiru_tang@fudan.edu.cn;](mailto:huiru_tang@fudan.edu.cn;) [wangsijia@sinh.ac.cn;](mailto:wangsijia@sinh.ac.cn;) [fengqian@fudan.edu.cn](mailto:fengqian@fudan.edu.cn)

**This PDF file includes:**

Figure S1 to S14

Legends for Table S1 to S18, Data S1 to S5

References (1 to 4)

**Other Supplementary Materials for this manuscript include the following:**

Table S1 to S18

Data S1 to S5


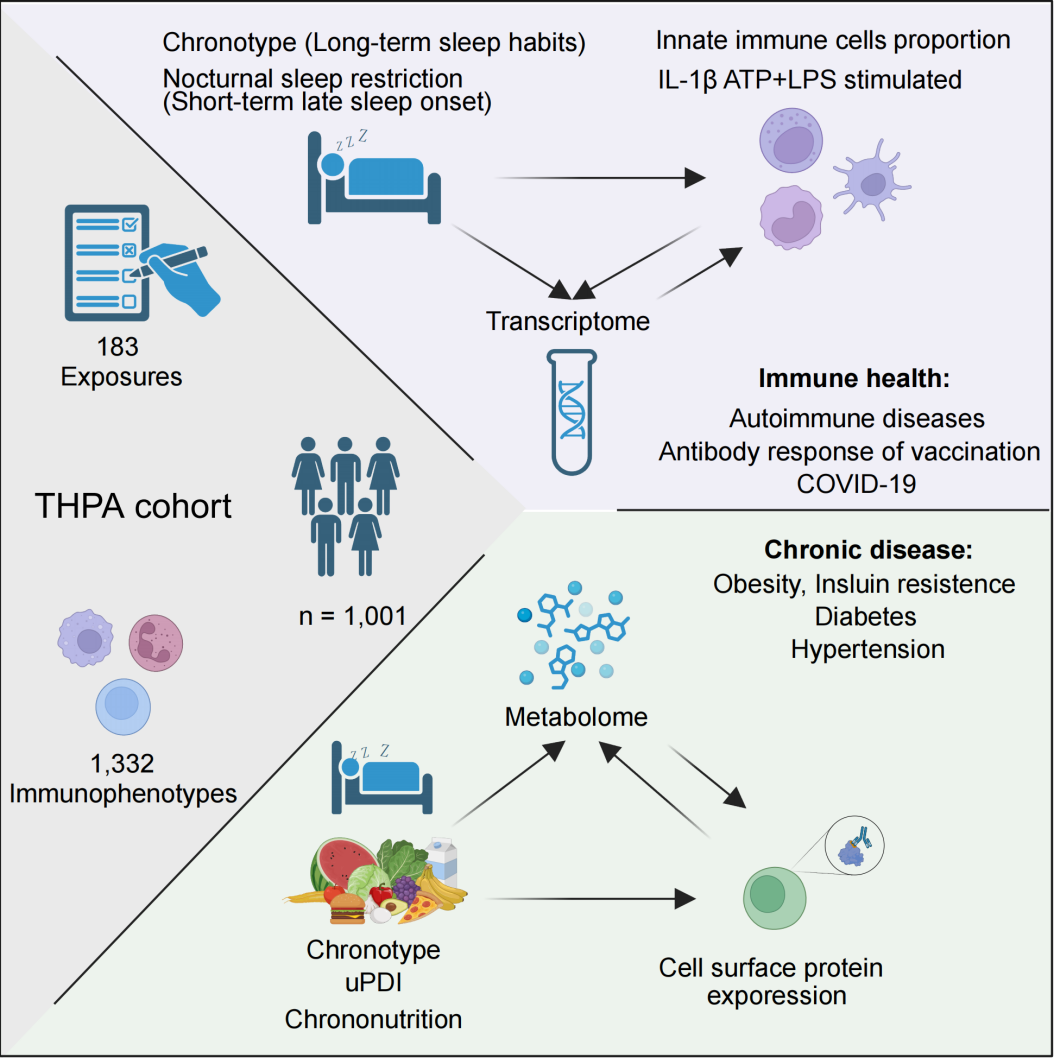


**Figure S1. Graphical abstract**

Graphical abstract of the whole study: Sleep and diet are the dominant exposures influence immunophenotypes, and they have mediation linkages with transcriptome and metabolome. The transcriptomic (T-) and metabolomic (M-) indexes can reflect immune health and chronic diseases.

uPDI, unhealthful plant-based diet index.


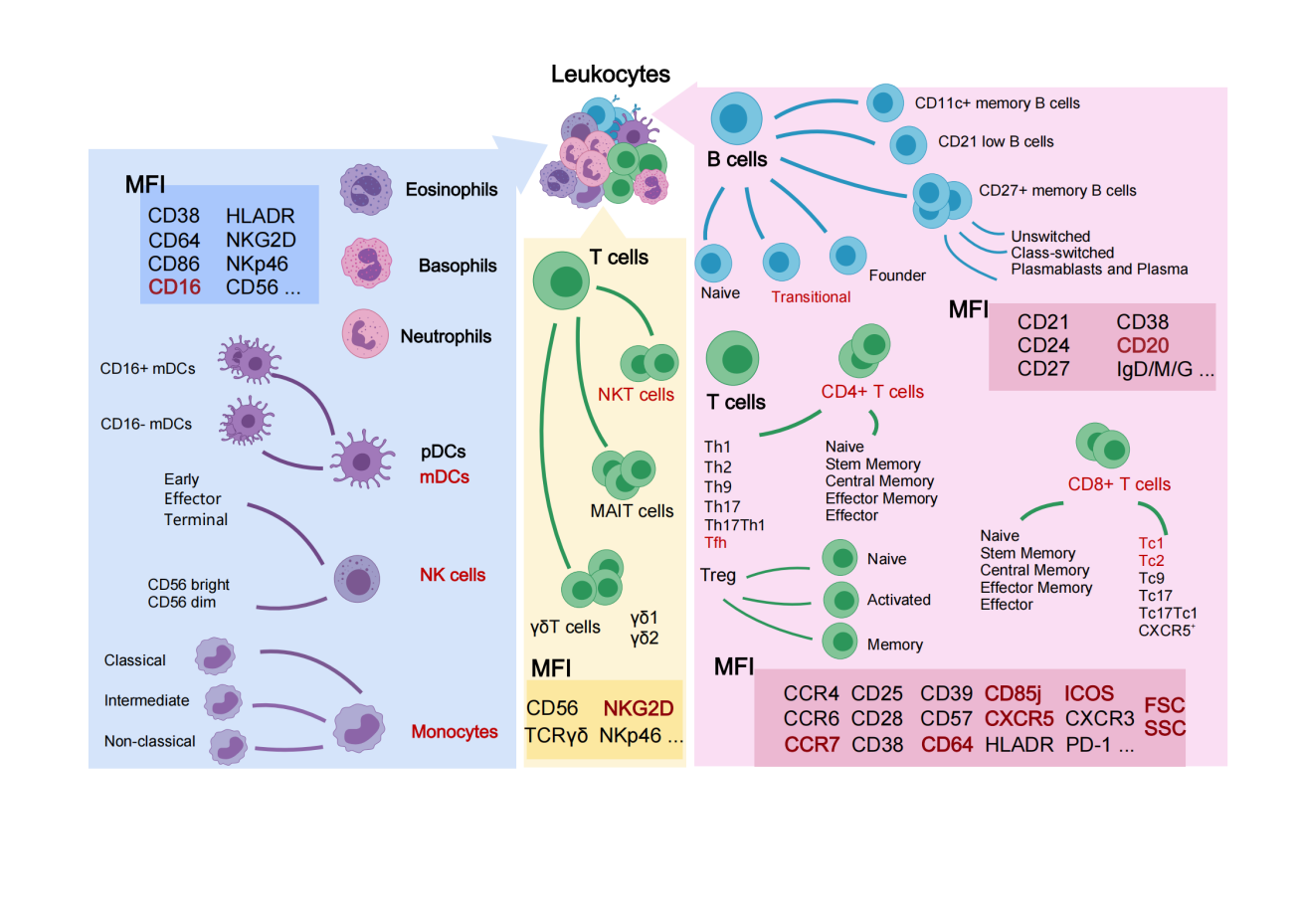


**Figure S2. Quantification of immune cells and cell surface markers measured in THPA cohort.** This figure was created with MedPeer (medpeer.cn). Strategy: Flow cytometry was used to quantify (as MFI) the expression of phenotypic markers of differentiation or activation in cells of various lineages or differentiation states (interconnecting lines), as well as to quantify the cells proportions (**Table S1**). A more detailed panel design can be found in our previous study[[1](#_ENREF_1)]. Red represents the immunophenotypes significantly affected by exposures.

MFI, Mean Fluorescence Intensity.


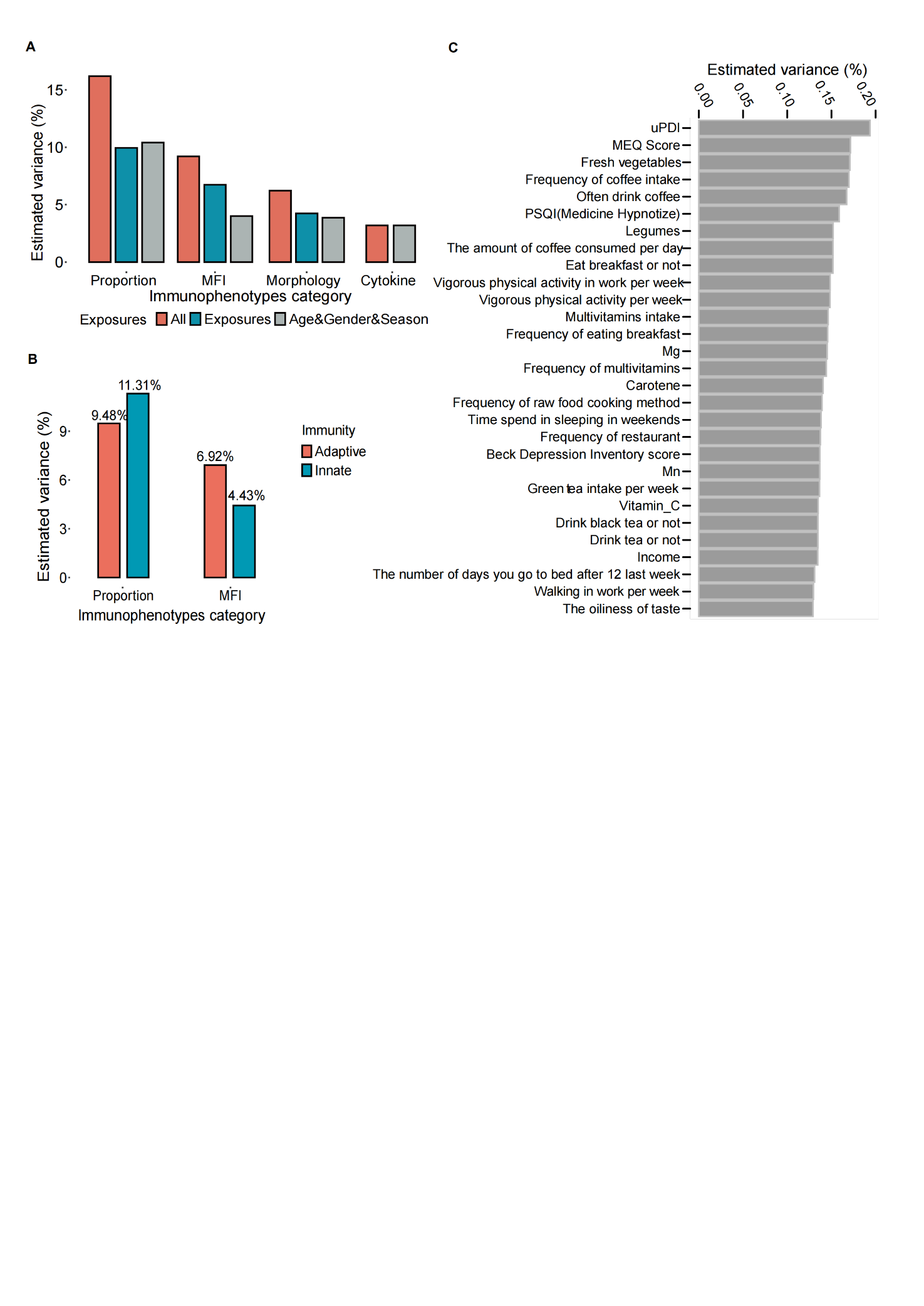


**Figure S3. Detailed information on immunome variation explained by exposures. (A)** Variance in different four datasets (Proportion, MFI, Morphology, Cytokine) explained by exposures, covariates and all these factors in multivariate PERMANOVA analysis. **(B)** Variance in innate immunophenotypes and adaptive immunophenotypes of proportion or MFI category explained by exposures in multivariate PERMANOVA analysis. **(C)** Variation in the immunophenotypes (corrected for age, gender, and season) explained by each exposure in PERMANOVA analysis (*P*-value < 0.05).

**
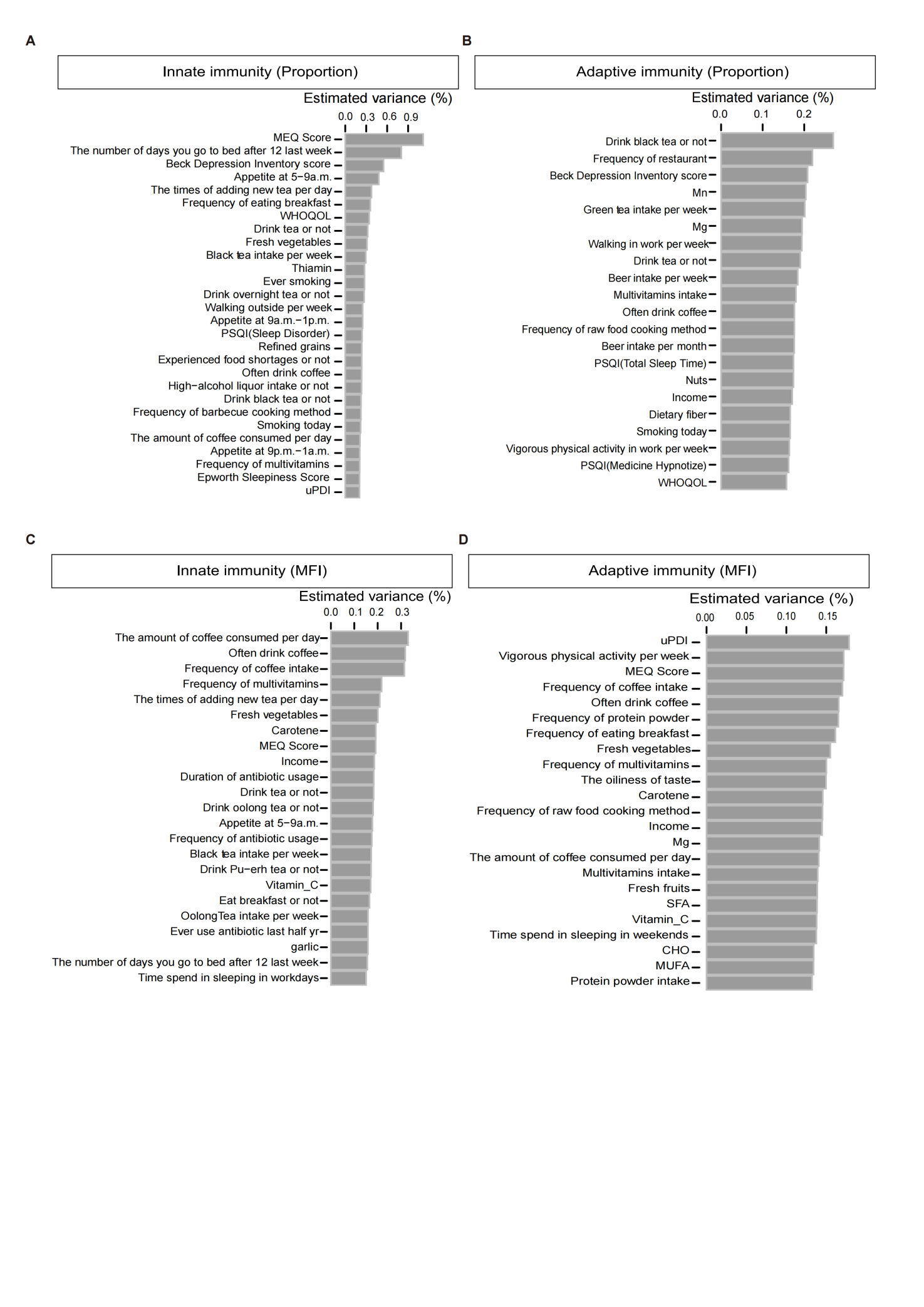
**

**Figure S4. Inter-individual variation in the different categories of immunophenotypes explained by each exposure. (A-D)** Variance in innate immunophenotypes (corrected for age, gender, and season) of proportion **(A)** or MFI **(C)** category and adaptive immunophenotypes (corrected for age, gender, and season) of proportion **(B)** or MFI **(D)** category explained by each exposure in PERMANOVA analysis (*P*-value < 0.05).

MFI, Mean Fluorescence Intensity.


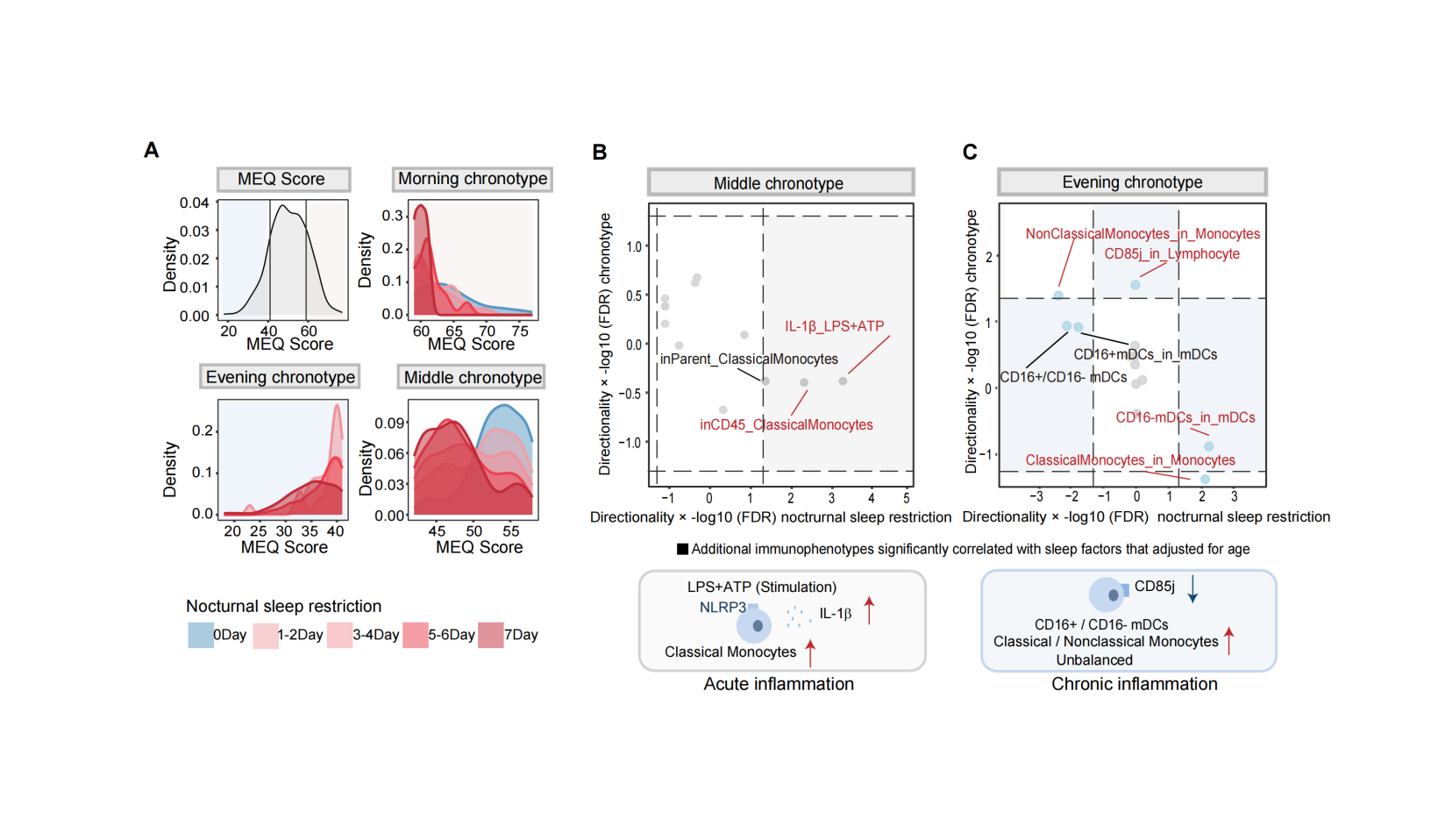


**Figure S5. Short-time late sleep onset differs from evening chronotype in causing acute inflammation of monocytes. (A)** MEQ score density plot of individuals with different nocturnal sleep restriction behaviors in different chronotype groups. **(B-C)** Immunophenotype associations with chronotype and nocturnal sleep restriction in middle **(B)** and evening chronotype **(C)** individuals. Each point represents an immunophenotype. The x-axis and y-axis denote the minus log10 FDR times the direction of the association in the general linear model of nocturnal sleep restriction and chronotype, respectively, adjusting for age, gender and season as confounders. Black dots represent additional immunophenotypes that were significantly correlated with sleep factors when we adjusted sleep factors for age.

MEQ, Morningness-Eveningness Questionnaire.


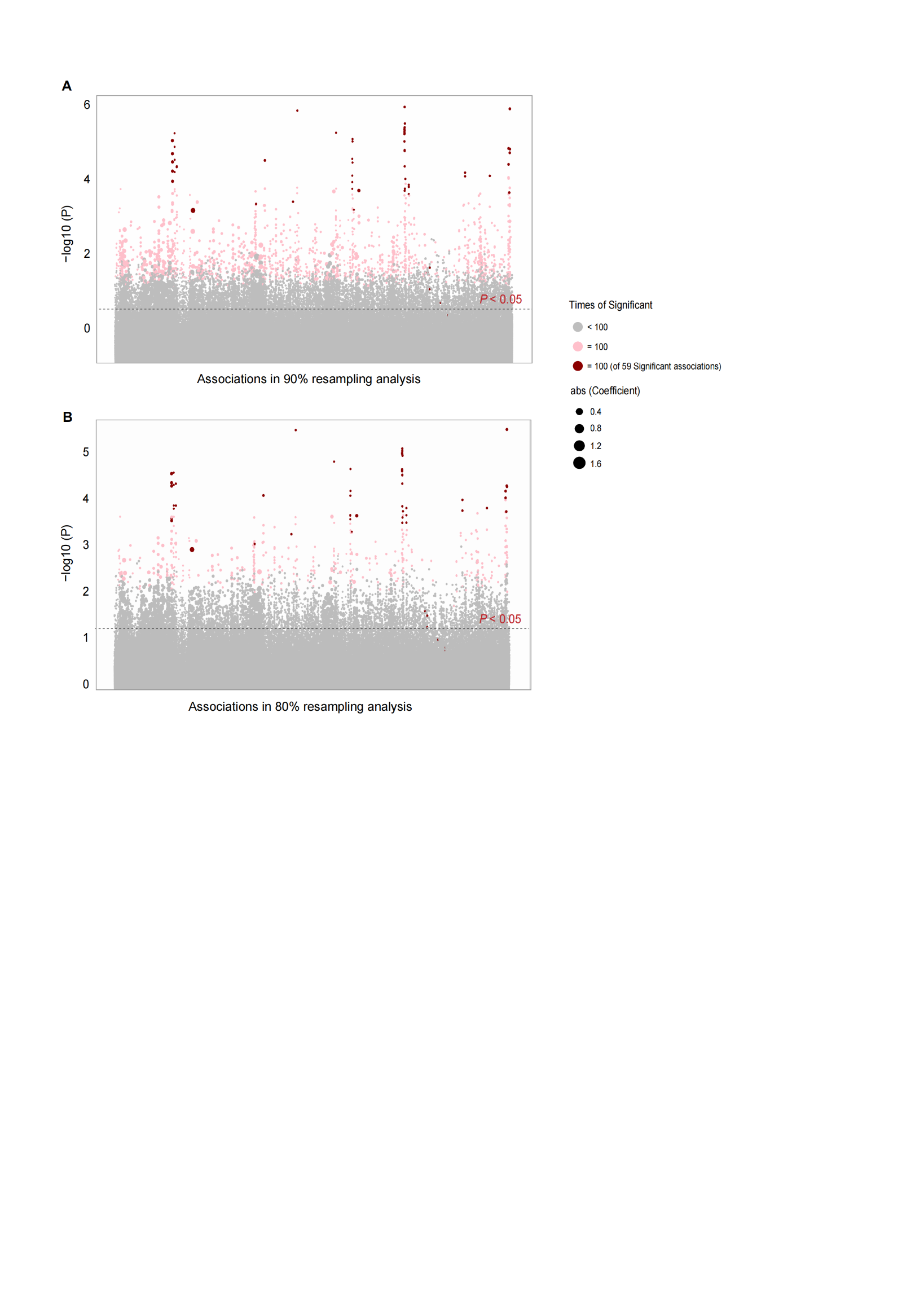


**Figure S6. Robustness of exposure effects on immunophenotypes. (A-B)** We randomly selected 90% **(A)** or 80% **(B)** of all the samples and performed statistical tests for each association. We repeated this 100 times and show the mean of the statistical significance (−log10 *P*-value) and coefficients.


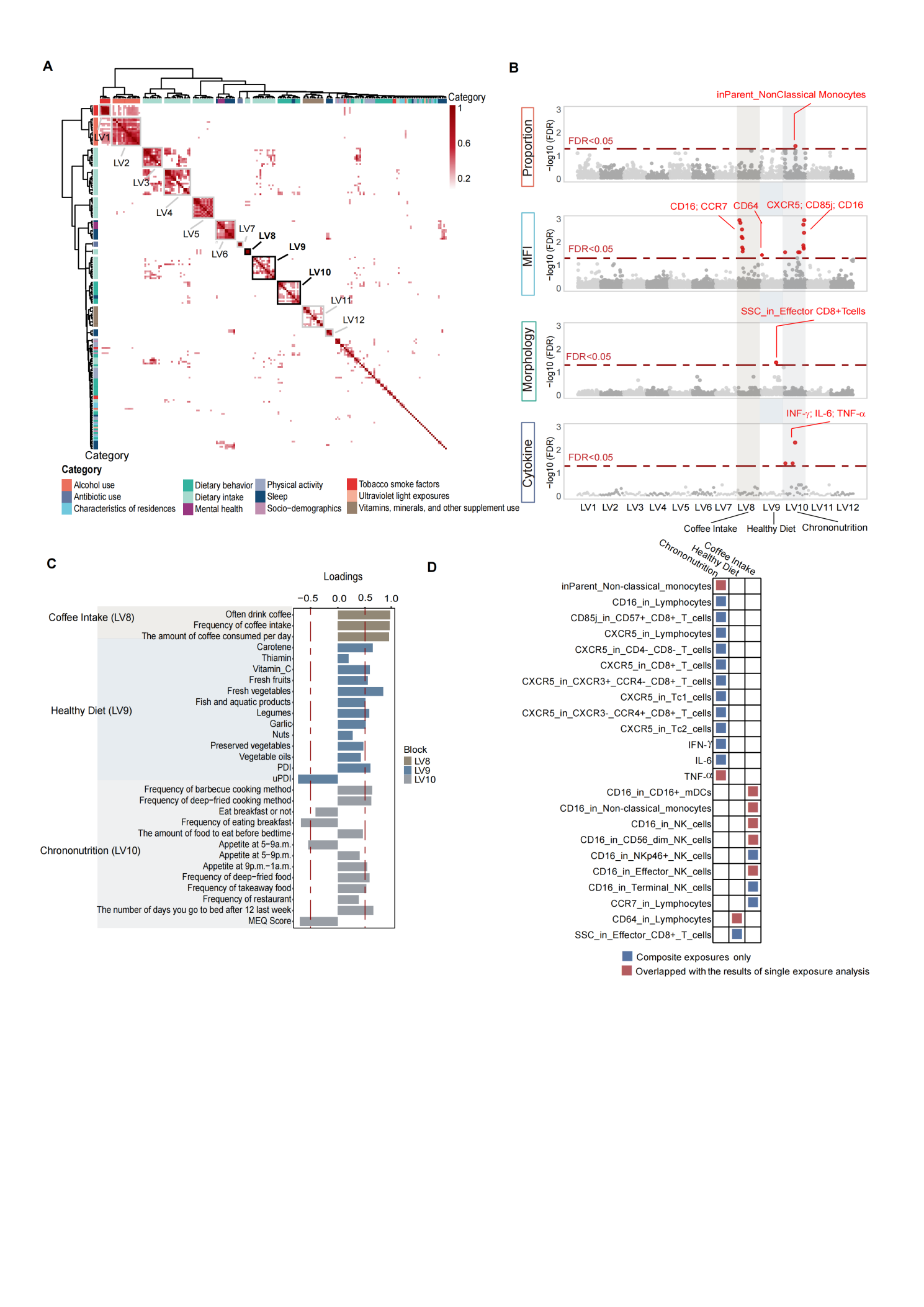


**Figure S7. Additional effects of composite exposures on the immunophenotypes. (A)** Ward clustering on Euclidean distances of Spearman’s correlation between exposures. **(B)** Summary of the associations between composite exposures and different categories of immunophenotypes. The y-axis indicates −log10 (FDR) of the regression coefficient for each of the association between composite exposure and immunophenotype. Red dots represent associations with significant coefficient. **(C)** The loadings of the single exposures in LV8 (Coffee Intake), LV9 (Healthy Diet) and LV10 (Chrononutrition). Dashed lines represent absolute loadings = 0.5. **(D)** Statistics on whether immunophenotypes significant in **B** are also significant in association studies of single exposures.

LV, Latent variable; SSC, side scatter.


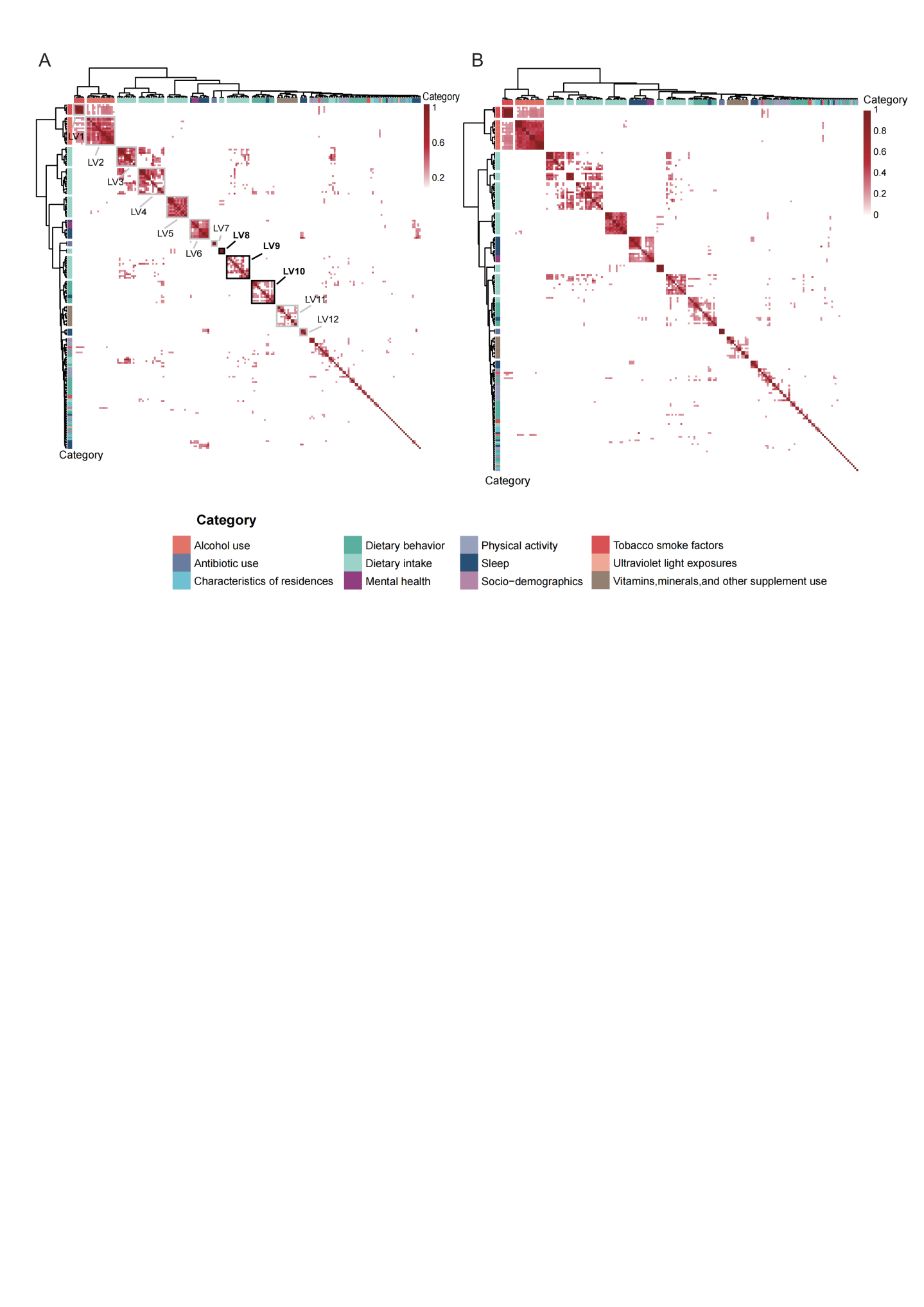


**Figure S8.** Ward clustering on Euclidean distances of Spearman’s correlation between exposures after **(A)** and before data imputation **(B)**.


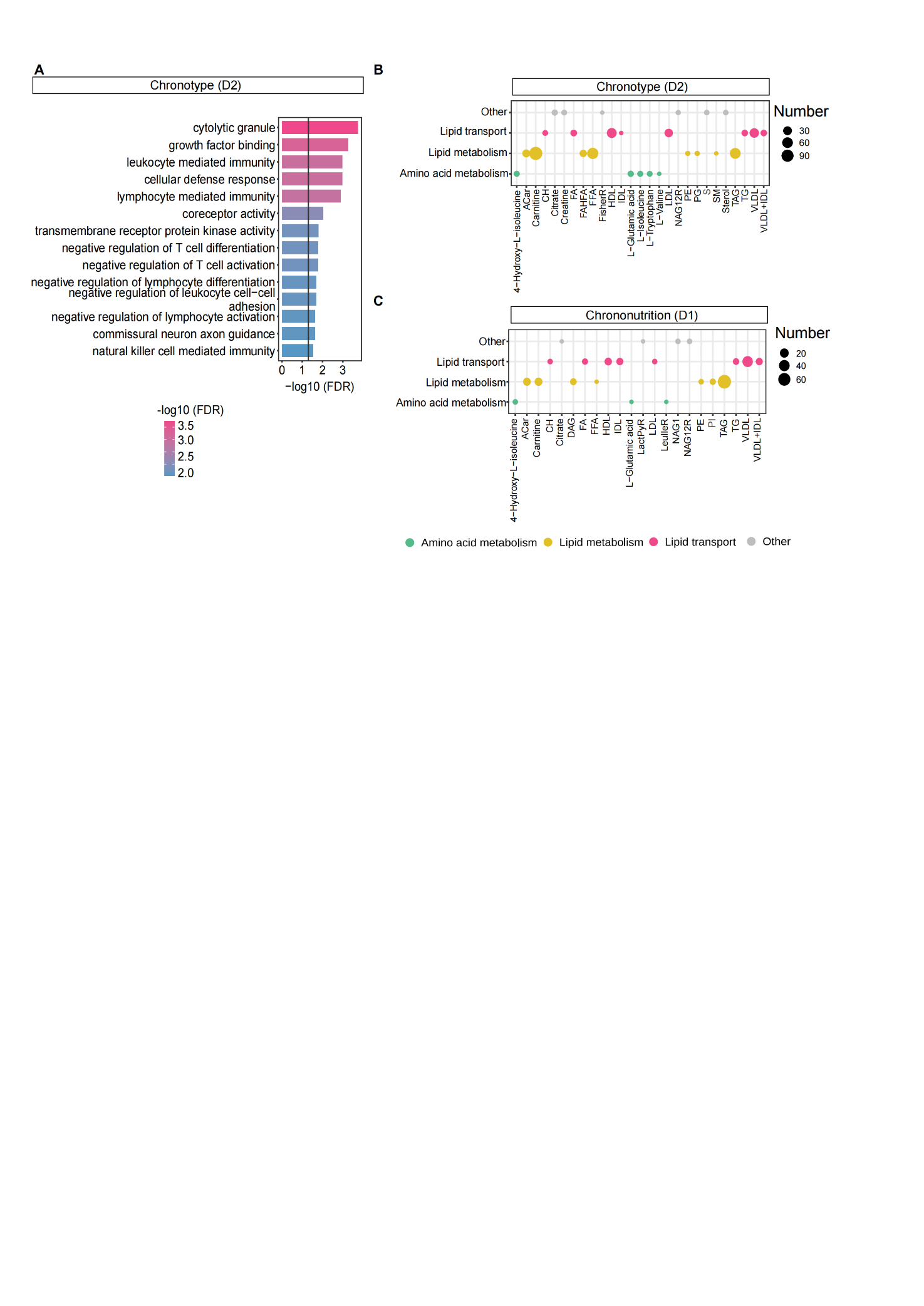


**Figure S9. Biological pathways of molecules that have significant ACME in mediations. (A)** Enriched GO pathways of genes with significant ACME in D2 mediations of chronotype. **(B-C)** Classification of lipids and metabolites with significant ACME in D1 mediations of chrononutrition (**B**), or in D2 mediations of chronotype (**C**).

ACME, average causal mediated effect; D1, Direction1; D2, Direction2.


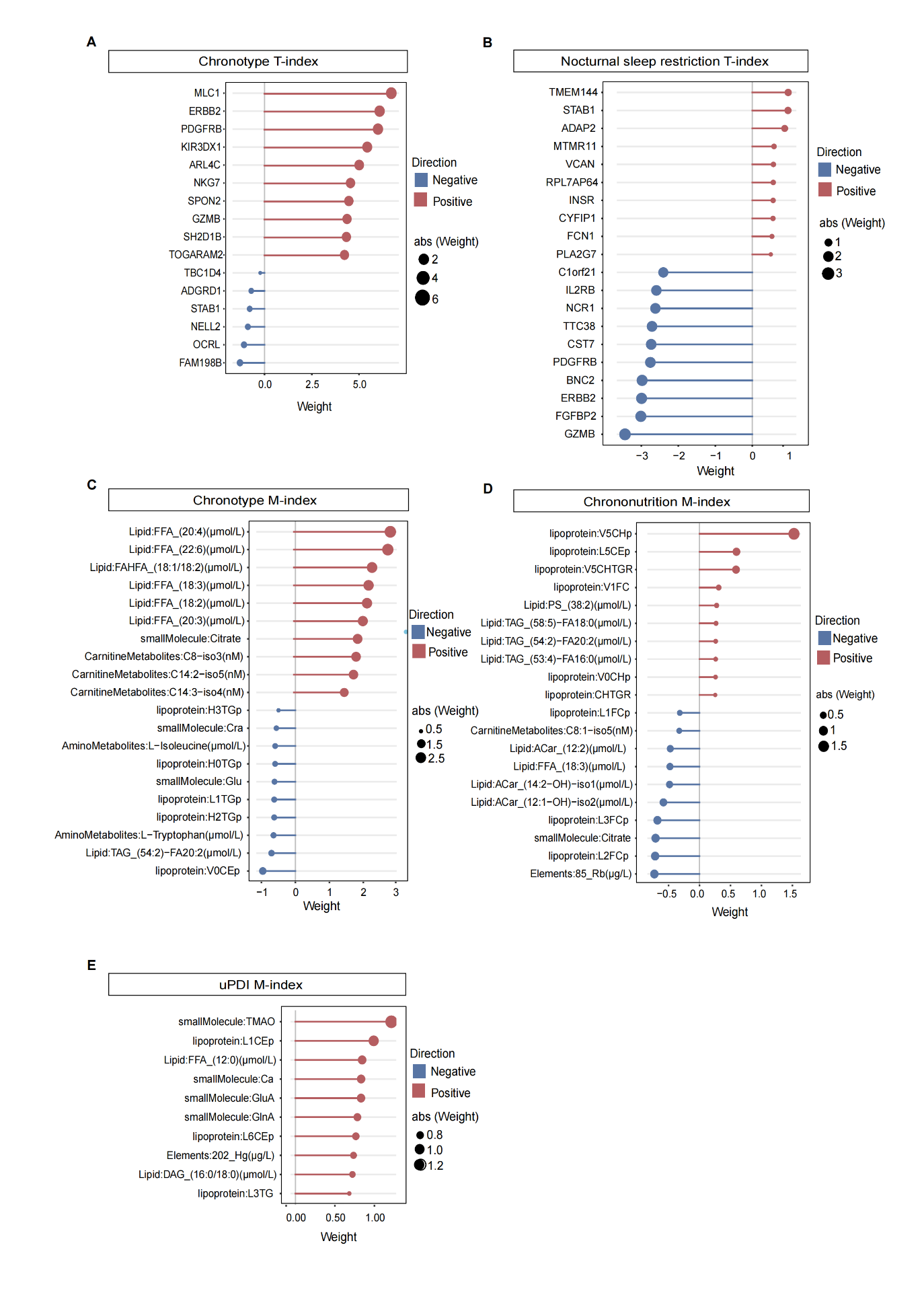


**Figure S10. The weights of the top-ranked genes, lipids and metabolites used to calculate the T- or M-indexes. (A-B)** Top-ranked genes of chronotype **(A)** or nocturnal sleep restriction **(B)** ranked by weighting coefficient. Only the top five genes with positive or negative weights are presented. **(C-E)** Top-ranked lipids and metabolites of chronotype **(C)**, chrononutrition **(D)** and uPDI **(E)** ranked by weighting coefficient. Only the top five lipids/metabolites with positive or negative weights are presented.


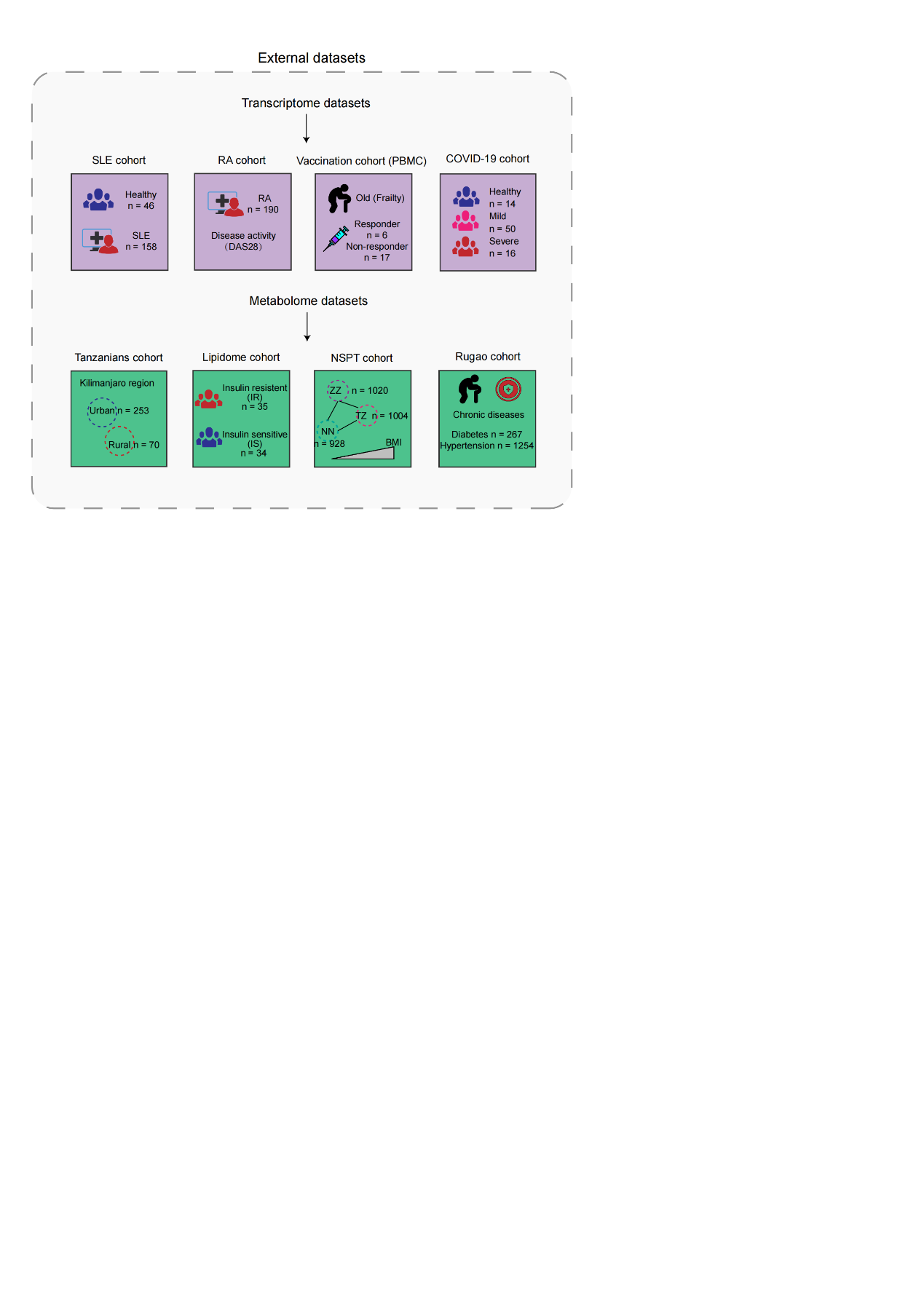


**Figure S11. Overview of 8 external datasets.** The 8 external datasets used to assess T- or M-indexes: The RA cohort were also referred to as the RA-MAP cohort. Detailed information on these datasets can be found in **Table S15**.

SLE, systemic lupus erythematosus; RA, rheumatoid arthritis; COVID-19, coronavirus disease 2019; NSPT, National Survey of Physical Traits; NN, Nanning, China; TZ, Taizhou, China; ZZ, Zhengzhou, China.


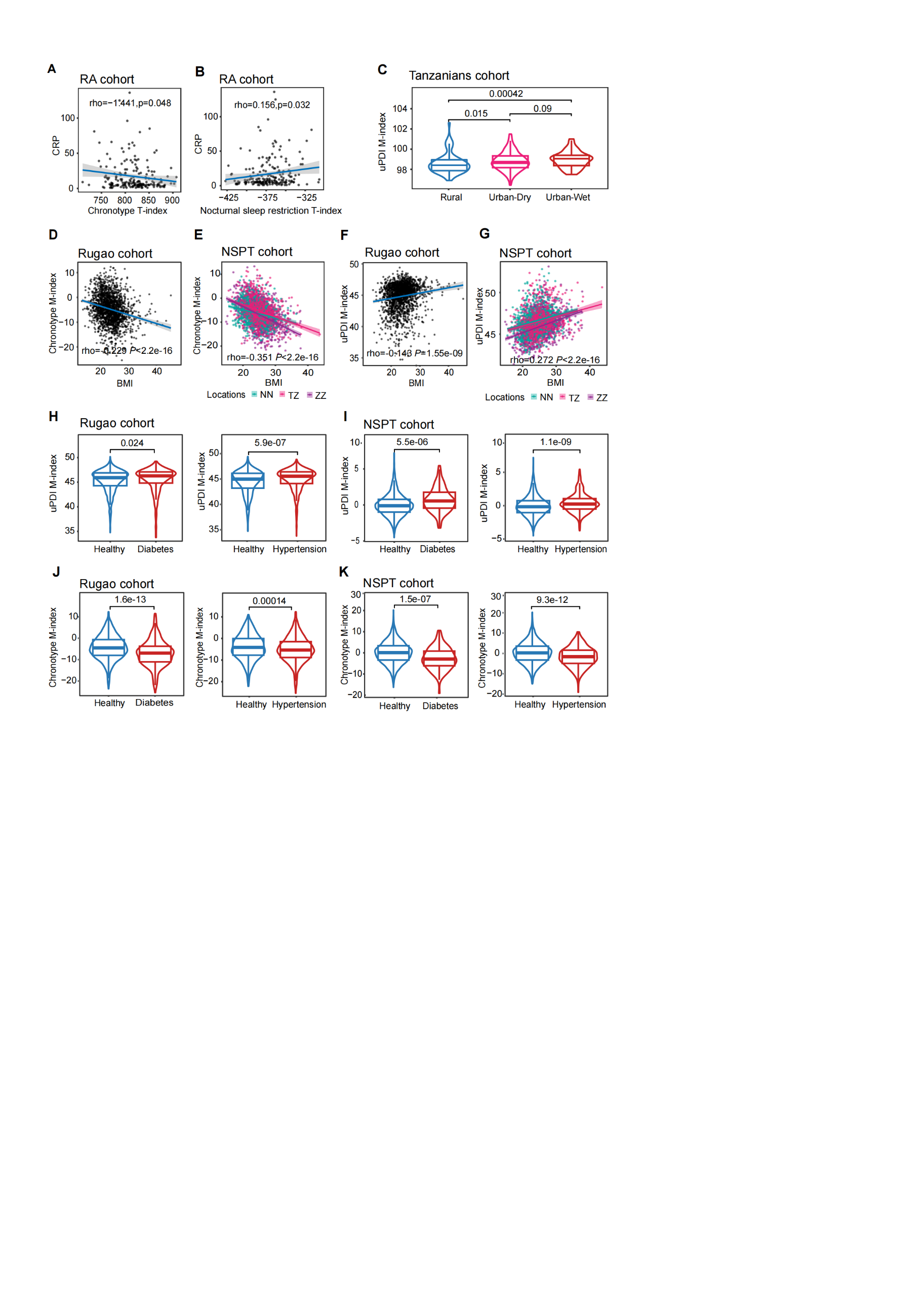


**Figure S12. Assessing T- or M-indexes in 8 external cohorts. (A-B)** Correlation between baseline (pre-treatment) chronotype **(A)** or nocturnal sleep restriction **(B)** T-index and CRP of 190 RA patients in the RA-MAP study[[2](#_ENREF_2)]. Pearson’s correlation and associated *P*-value are shown. The shaded area represents the 95% CI. **(C)** uPDI M-indexes stratified by dry-season urban, wet-season urban and rural in a Tanzanians cohort[[3](#_ENREF_3)]. *P*-values from paired, two-tailed Wilcoxon test are shown (n = 168 urban residents in dry season, 79 urban residents in wet season and 69 rural residents). **(D-E)** Scatterplot with trend line showing the positive correlation between chronotype M-index and BMI in the Rugao cohort[[4](#_ENREF_4)] **(D)** and NSPT cohort (3 locations; **E**). Pearson’s correlation and associated *P*-value are shown. The shaded area represents the 95% confidence interval (95% CI). **(F-G)** Scatterplot with trend line showing the positive correlation between uPDI M-index and BMI in the Rugao cohort[[4](#_ENREF_4)] **(F)** and NSPT cohort (3 locations; **G**). Global Pearson’s correlation and associated *P*-value are shown. The shaded area represents the 95% confidence interval (95% CI). **(H-I)** uPDI M-indexes classified by chronic diseases in the Rugao cohort[[4](#_ENREF_4)] **(H)** and NSPT cohort **(I)**. Indexes were adjusted for locations. *P*-values from paired, two-tailed Wilcoxon test are shown. **(J-K)** Chronotype M-indexes classified by chronic diseases in the Rugao cohort[[4](#_ENREF_4)] **(J)** and NSPT cohort **(K)**. Indexes were adjusted for locations. P-values from paired, two-tailed Wilcoxon test are shown.

Urban-Dry, Urban-Dry season; Urban-Wet, Urban-Wet season.


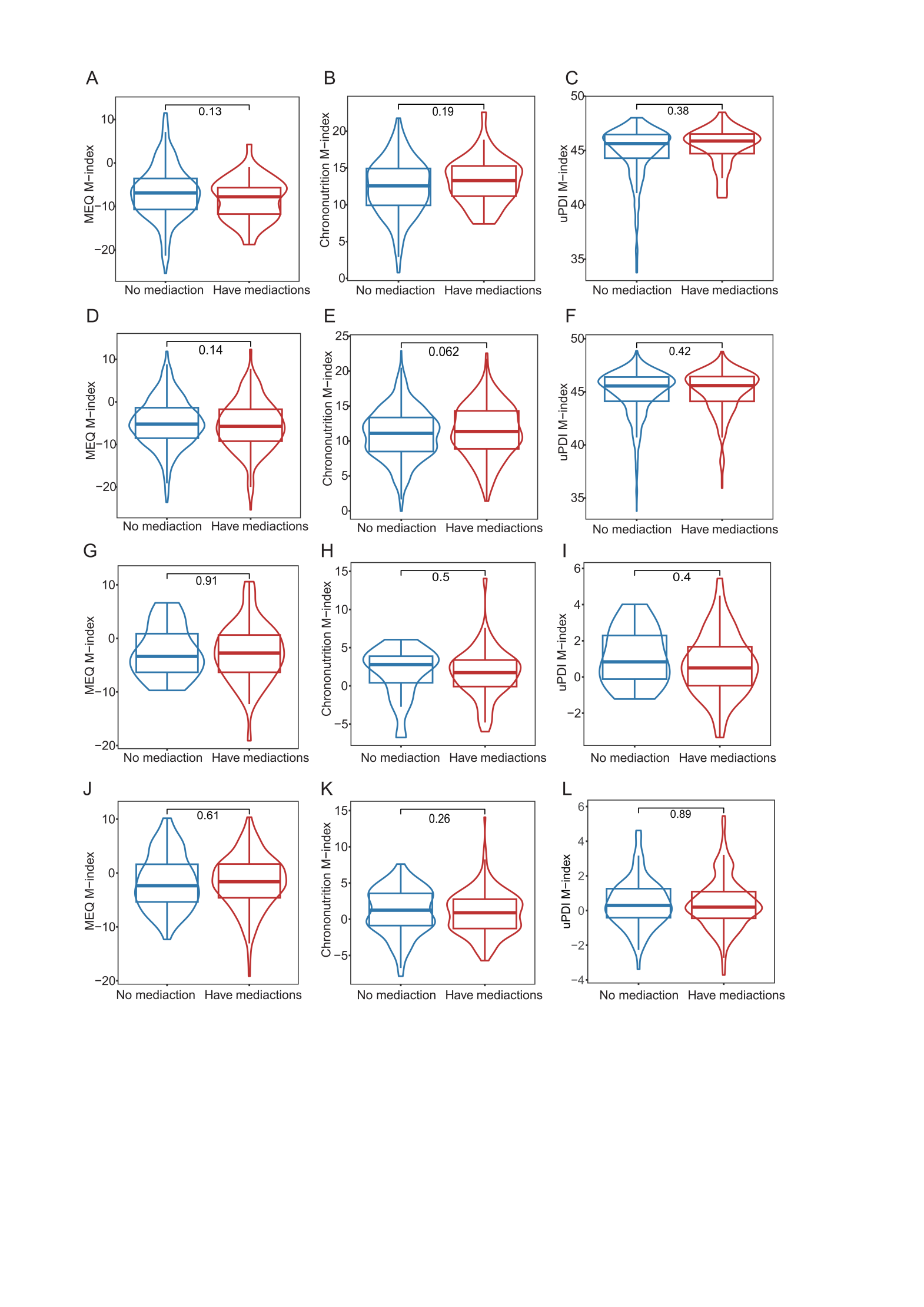


**Figure S13. M-indexes in the patient subgroups (with or without medications). (A-F)** M-indexes of diabetic **(A-C)** or hypertensive **(D-F)** patients with or without medications in Rugao cohort. **(G-L)** M-indexes of diabetic **(G-I)** or hypertensive **(J-L)** patients with or without medications in NSPT cohort.


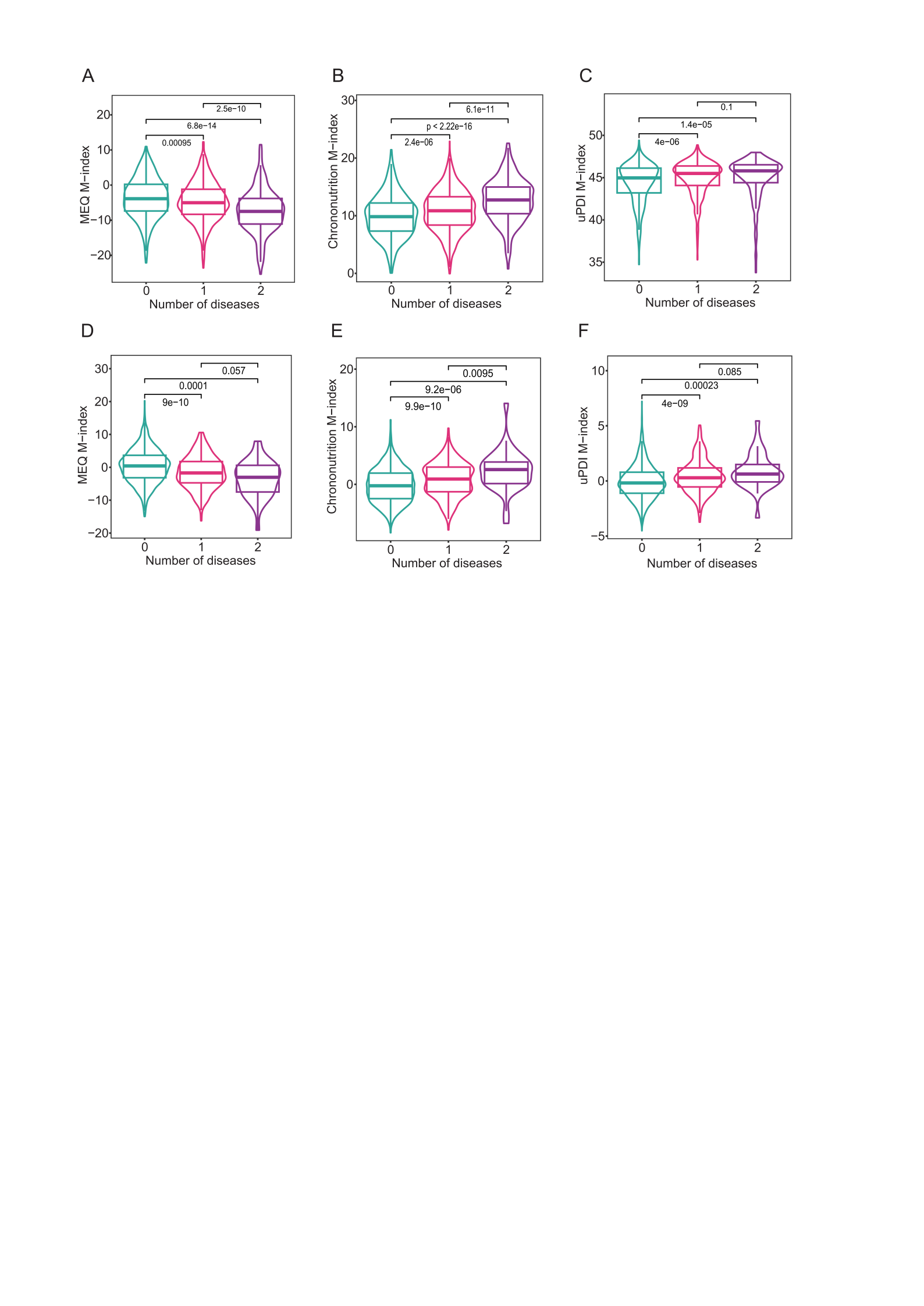


**Figure S14. Differences of M-indexes among people without or with one/two diseases (diabetes, hypertension). (A-C)** The M-index differences in Rugao cohort. **(D-F)** The M-index differences in NSPT cohort. The M-indexes were adjusted for locations.

**Legends for Tables S1 to S18, Data S1 to S5**

**Supplementary Tables 1 – 18**:

**Table S1**. Summary of 1,332 annotated immunophenotypes and their associations with age, gender and season

**Table S2**. 183 exposures involved in the analysis

**Table S3**. Significant associations between exposures and immunophenotypes in THPA cohort (FDR < 0.05; before and after the data imputation)

**Table S4**. 59 associations between exposures and immunophenotypes in resampling analysis

**Table S5**. Sensitivity analysis of exposure-immunophenotype associations (BMI, COVID-19 vaccination, COVID-19 and genetic PCs as covariates, respectively)

**Table S6**. 59 associations in different gender or age group

**Table S7**. Significant associations between exposures and immunophenotypes in female, male or 20-39 years old group (FDR < 0.05)

**Table S8**. The composite exposures in THPA cohort

**Table S9**. Significant associations between composite exposures and immunophenotypes in THPA cohort (FDR < 0.05)

**Table S10**. Direction1 meidation linkages of exposures, transcriptome and immunophenotypes

**Table S11**. Direction2 meidation linkages of exposures, immunophenotypes and transcriptome

**Table S12**. Direction1 meidation linkages of exposures, metabolome and immunophenotypes

**Table S13**. Direction2 meidation linkages of exposures, immunophenotypes and metabolome

**Table S14**. The weights of transcripts, lipids or metabolites that used to construct T-indexes or M-indexes

**Table S15**. Reference of 8 external datasets

**Table S16**. 18 health-related indicators involved in the analysis

**Table S17**. Associations between health-related indicators and T- or M-indexes

**Table S18**. Associations between health status and T- or M-indexes after correcting for covariates

**Data S1 to S5**:

**Data S1**. Source data of Figure 1

**Data S2**. Source data of Figure 2

**Data S3**. Source data of Figure 3

**Data S4**. Source data of Figure 4

**Data S5**. Source data of Figure 5

**References**

1. Gao, J., et al., *Deep Immunophenotyping of Human Whole Blood by Standardized Multi-parametric Flow Cytometry Analyses.* Phenomics, 2023. 3(3): p. 309-328.

2. Consortium, R.-M., *RA-MAP, molecular immunological landscapes in early rheumatoid arthritis and healthy vaccine recipients.* Sci Data, 2022. 9(1): p. 196.

3. Temba, G.S., et al., *Urban living in healthy Tanzanians is associated with an inflammatory status driven by dietary and metabolic changes.* Nat Immunol, 2021. 22(3): p. 287-300.

4. Pu, Y., et al., *Gut microbial features and circulating metabolomic signatures of frailty in older adults.* Nat Aging, 2024. 4(9): p. 1249-1262.
