# Supplementary figures and images for: The Influence of Sleep and Diet on Human Peripheral Immunity and Chronic Health Conditions
Source: Research (Wash D C). 2026 Feb 19;9:1081. doi: 10.34133/research.1081 (PMC12943795; doi:10.34133/research.1081)

A

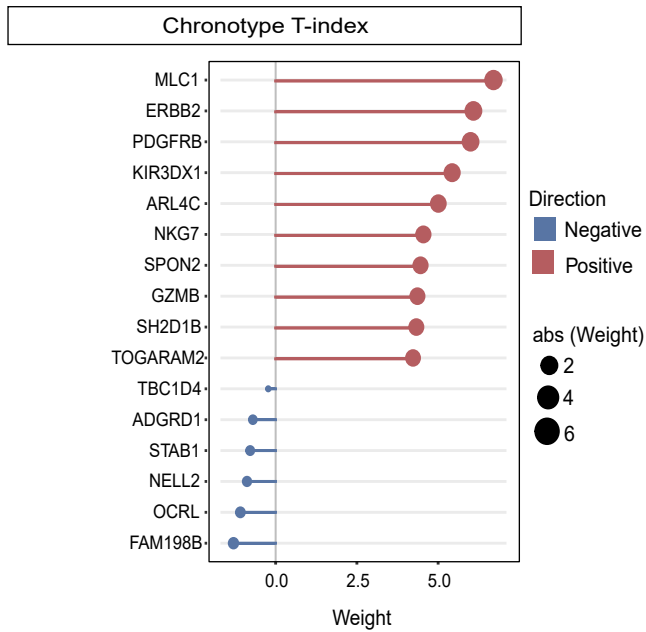

B

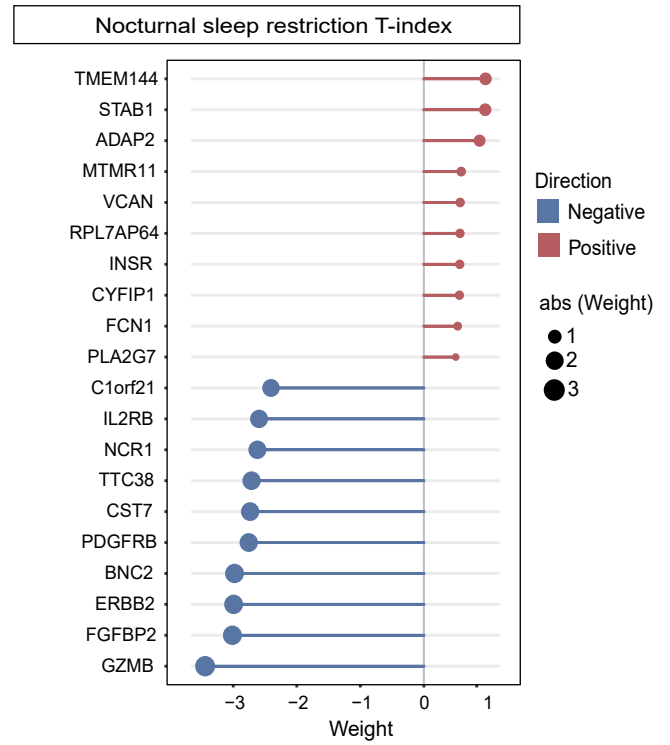

C

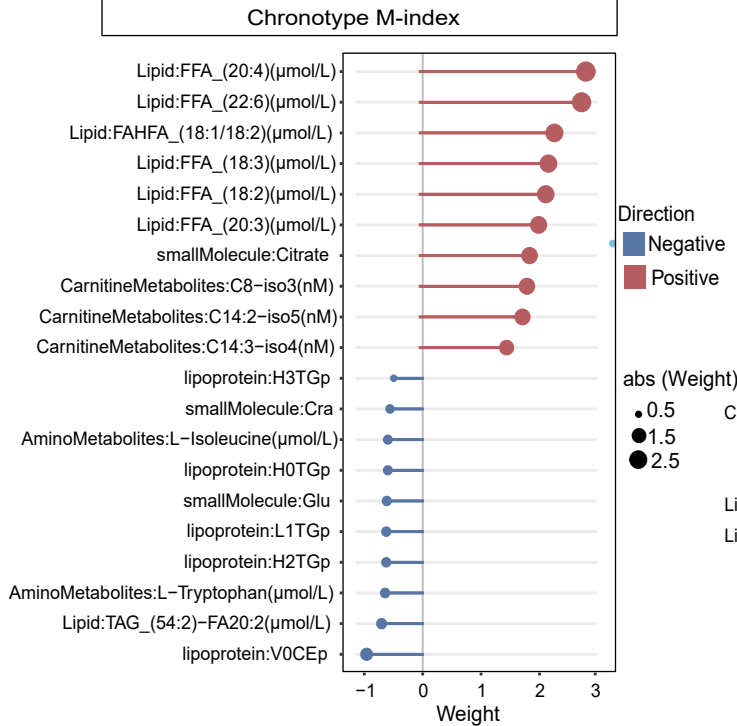

D

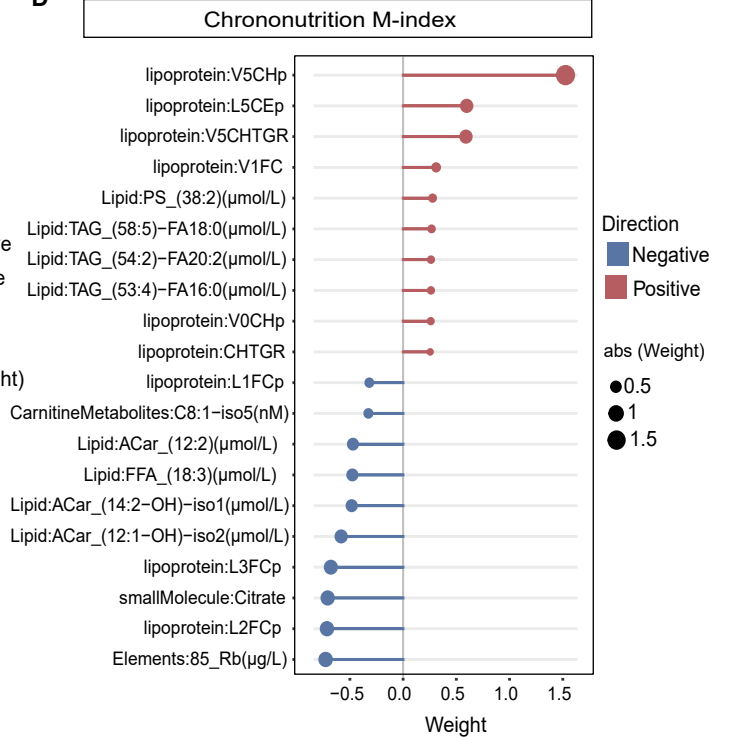

E

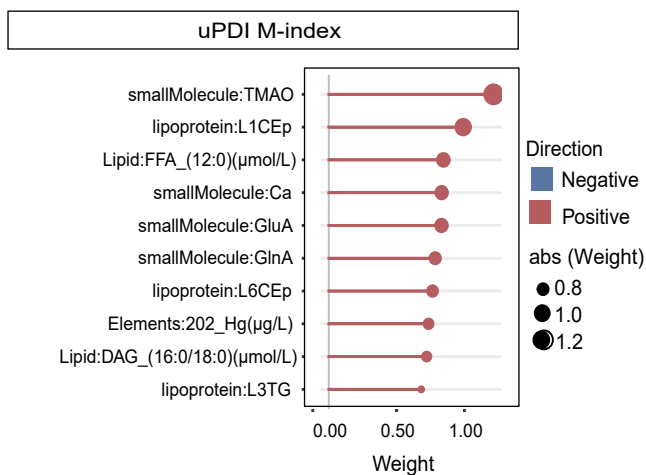

Supplement: Supplementary 1 — Figs. S1 to S14 Tables S1 to S18 Data S1 to S5 [file research.1081.f1.zip › Fig S10.pdf]

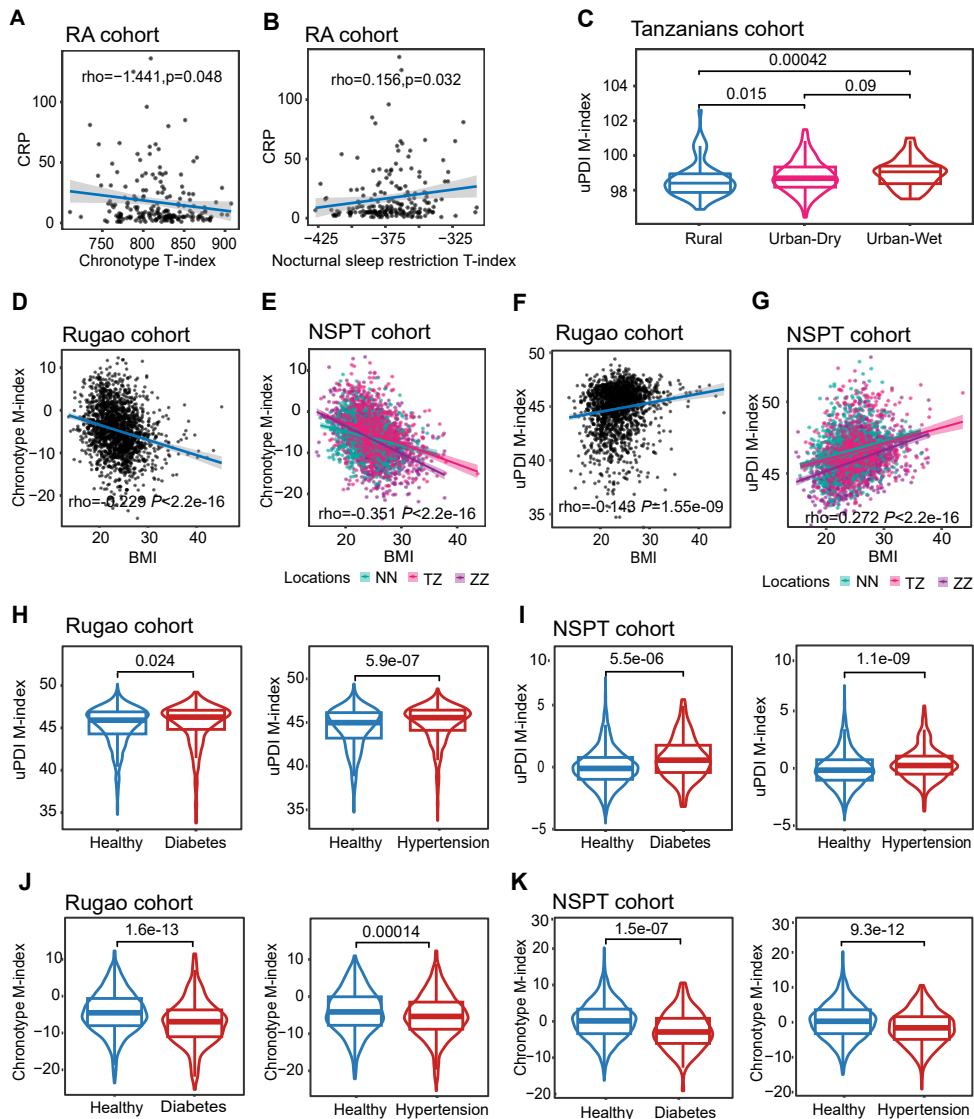

Supplement: Supplementary 1 — Figs. S1 to S14 Tables S1 to S18 Data S1 to S5 [file research.1081.f1.zip › Fig S12.pdf]

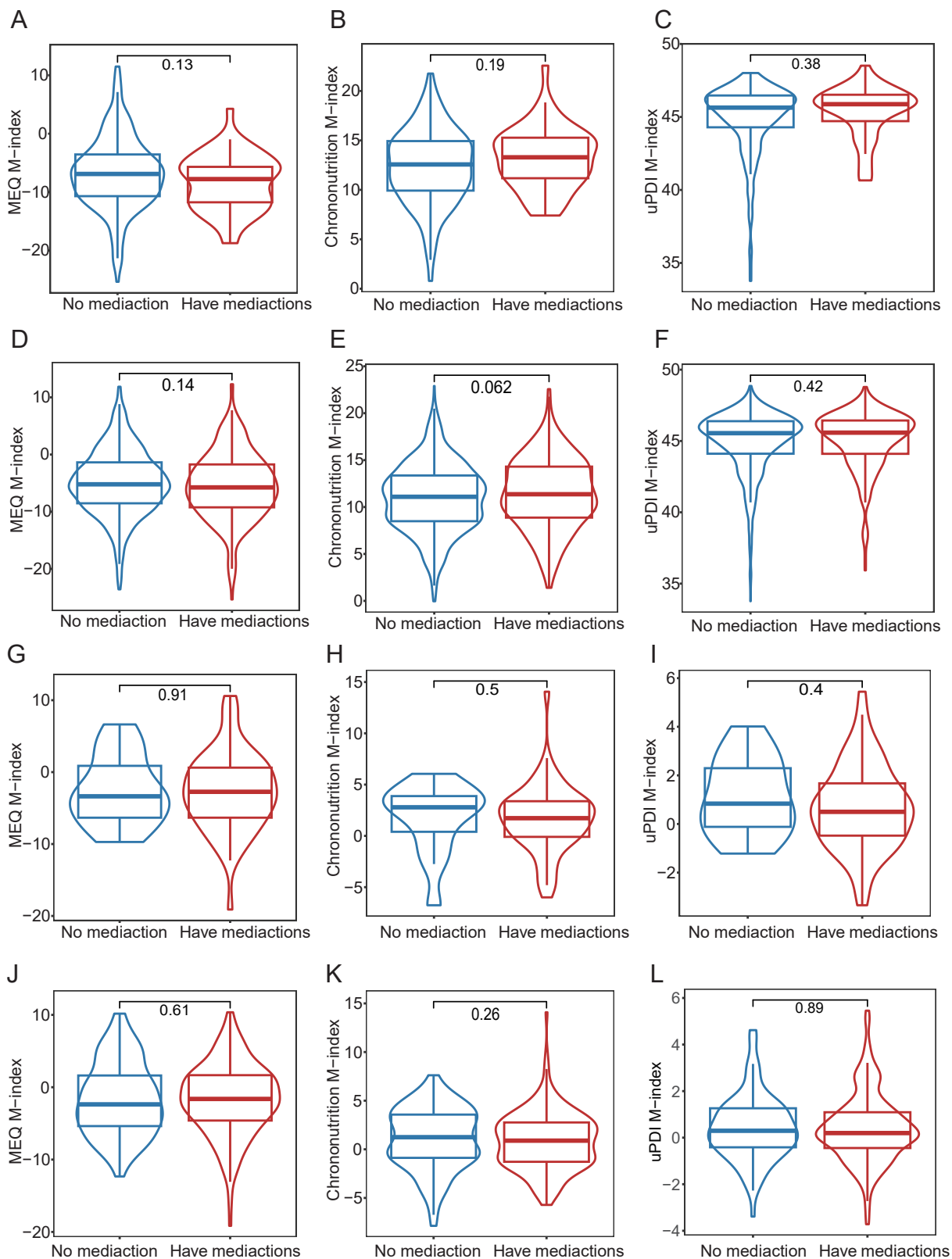

Supplement: Supplementary 1 — Figs. S1 to S14 Tables S1 to S18 Data S1 to S5 [file research.1081.f1.zip › Fig S13.pdf]

**A**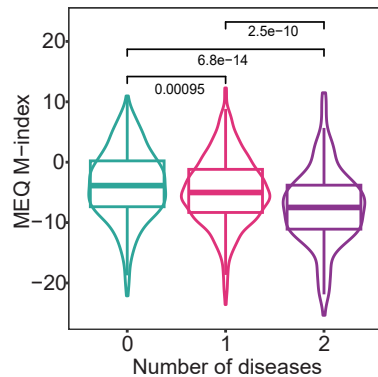**B**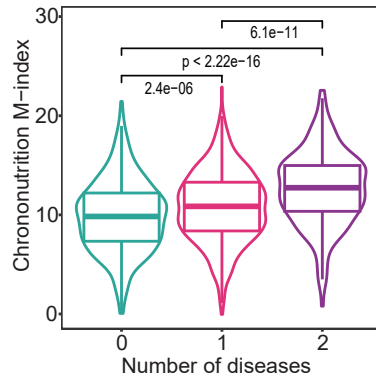**C**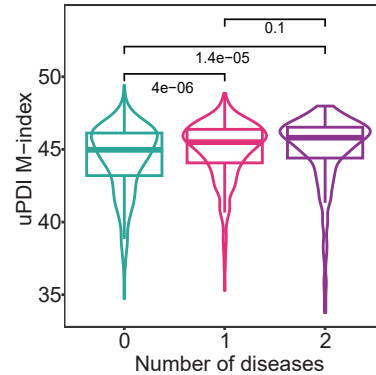**D**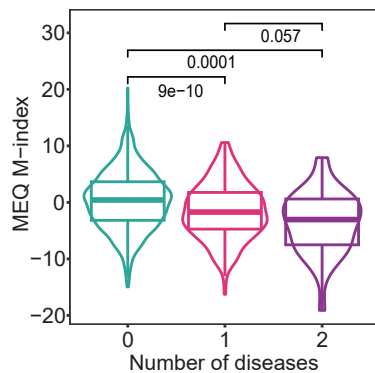**E**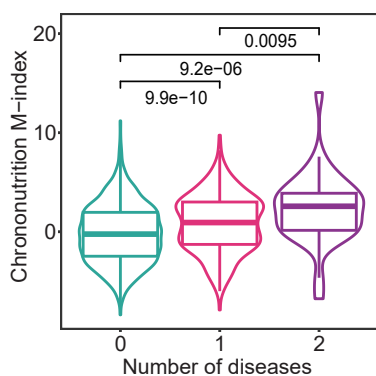**F**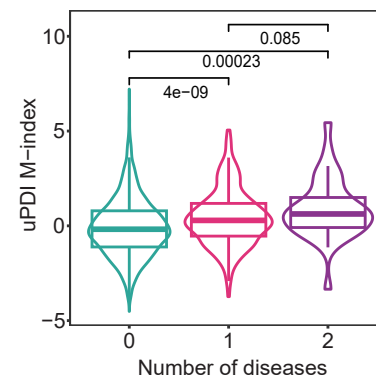

Supplement: Supplementary 1 — Figs. S1 to S14 Tables S1 to S18 Data S1 to S5 [file research.1081.f1.zip › Fig S14.pdf]

# Leukocytes

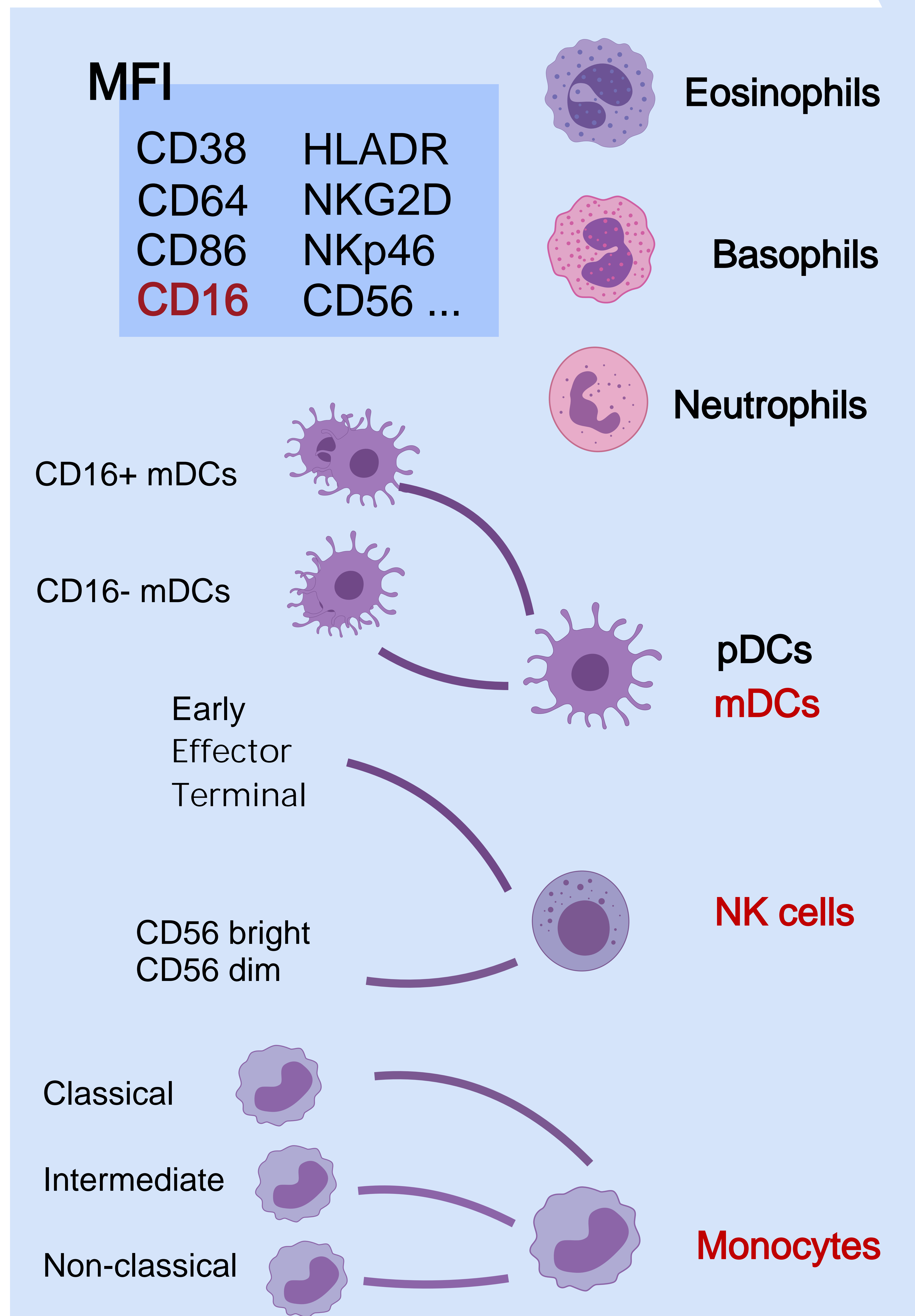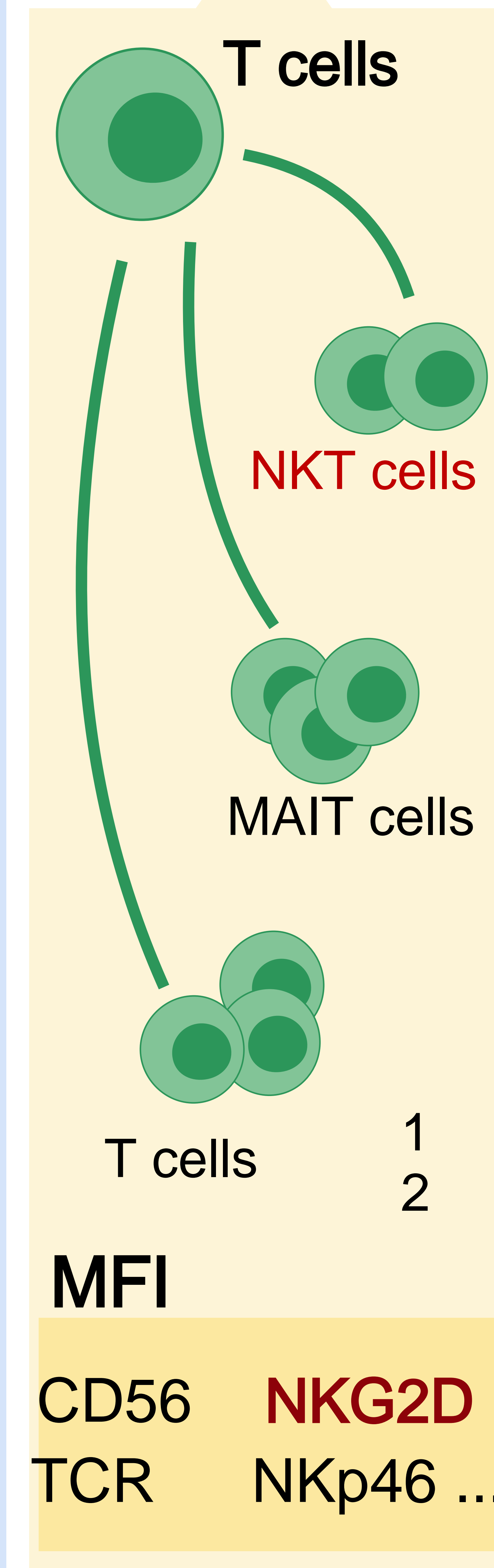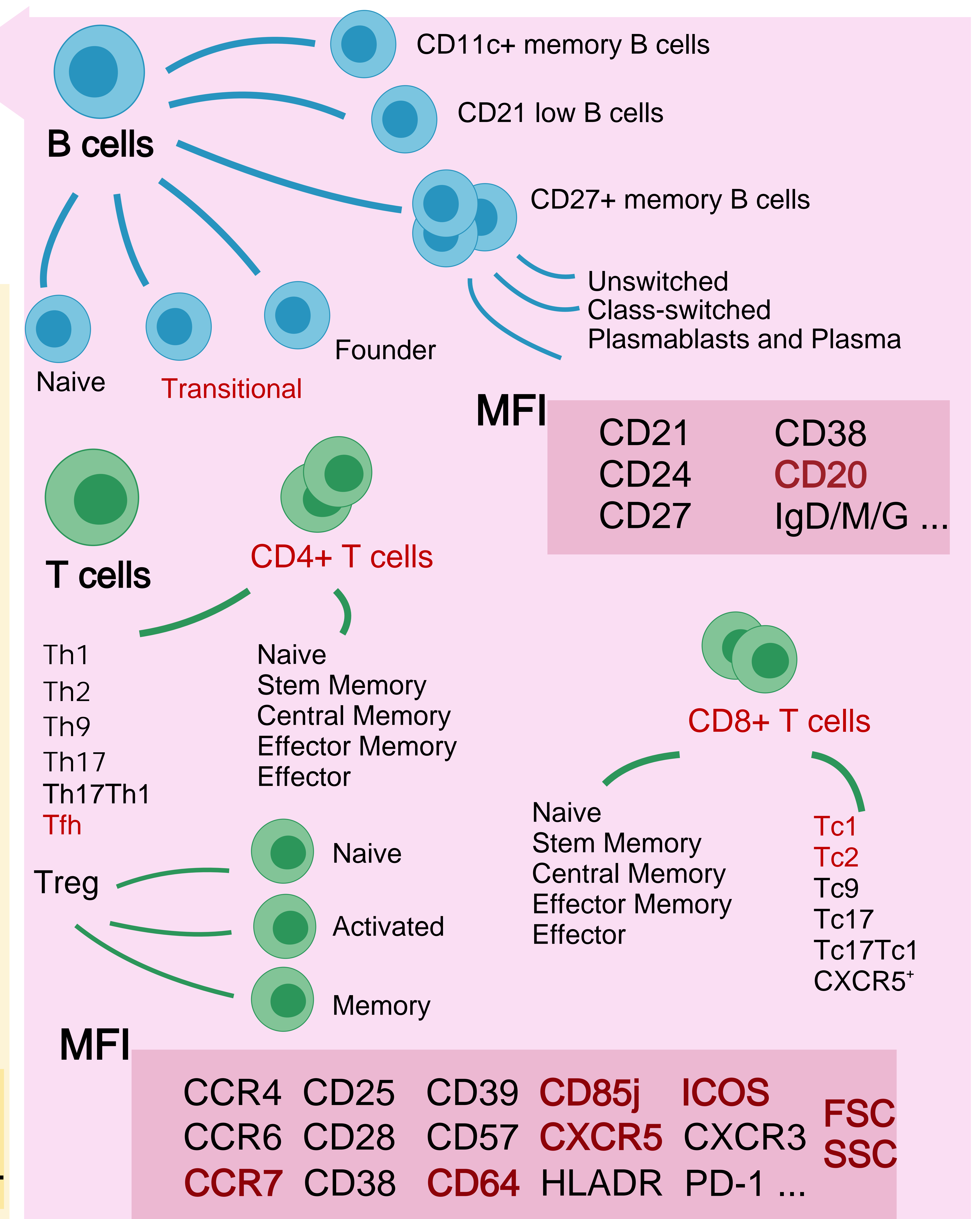

Supplement: Supplementary 1 — Figs. S1 to S14 Tables S1 to S18 Data S1 to S5 [file research.1081.f1.zip › Fig S2.pdf]

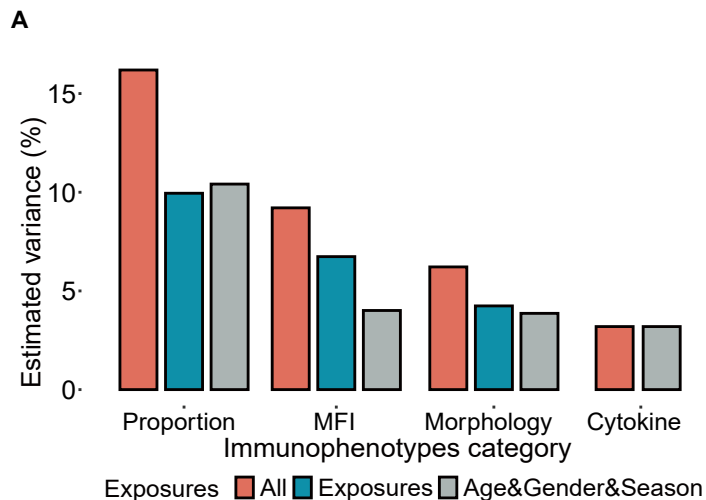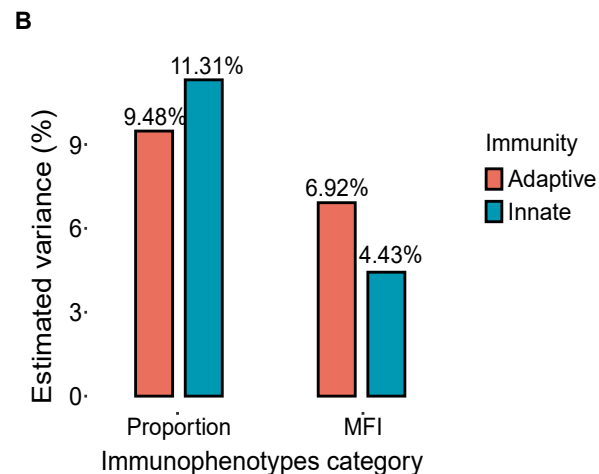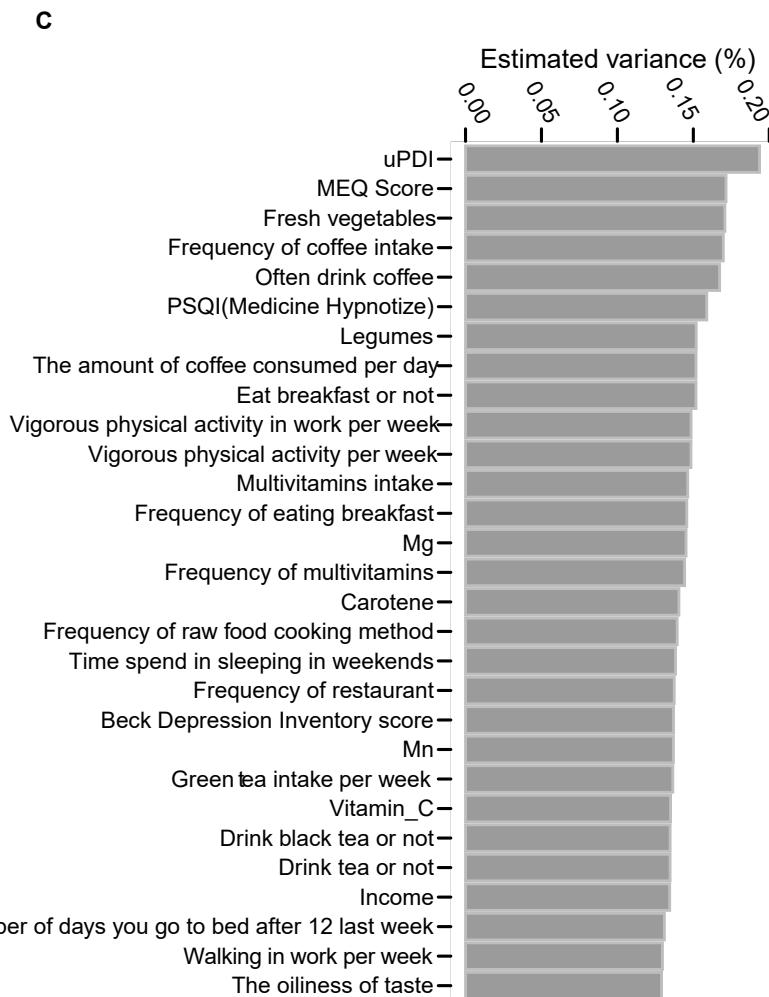

Supplement: Supplementary 1 — Figs. S1 to S14 Tables S1 to S18 Data S1 to S5 [file research.1081.f1.zip › Fig S3.pdf]

A

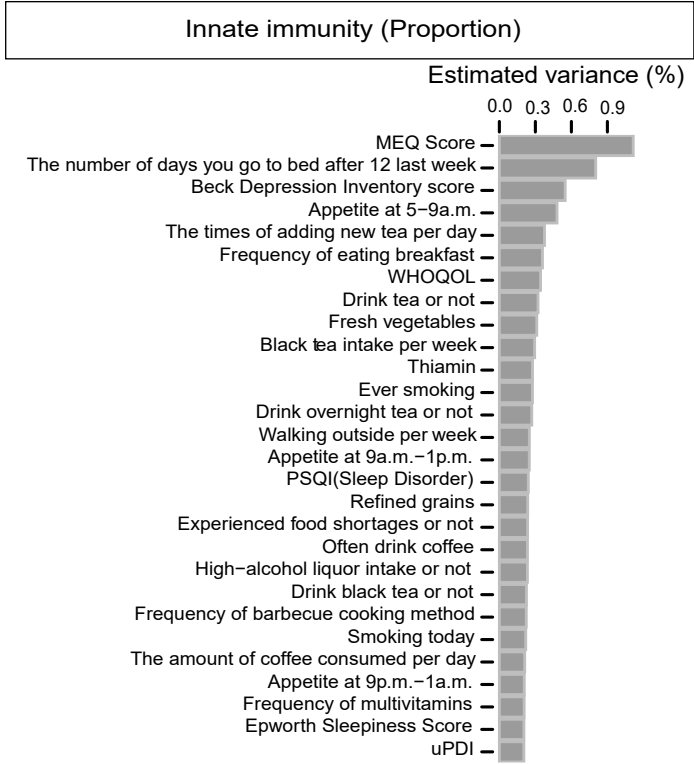

B

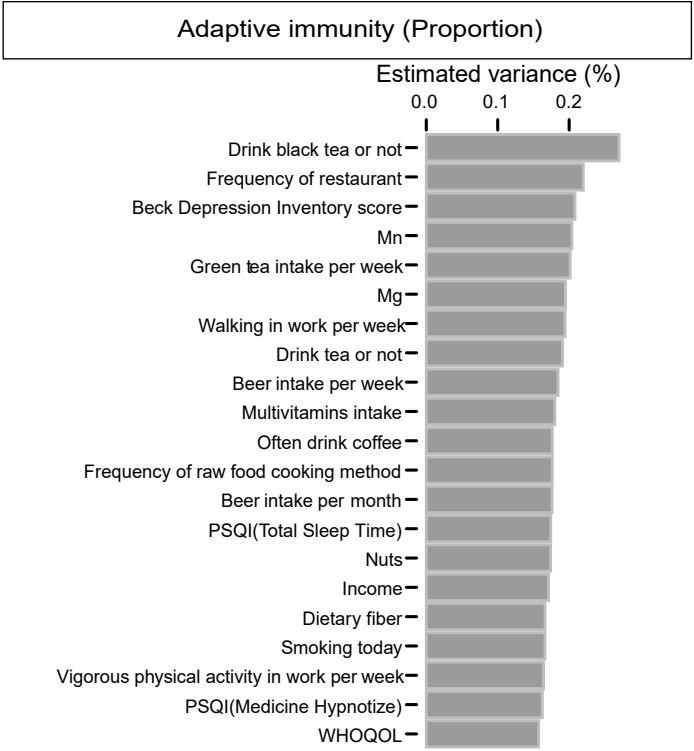

C

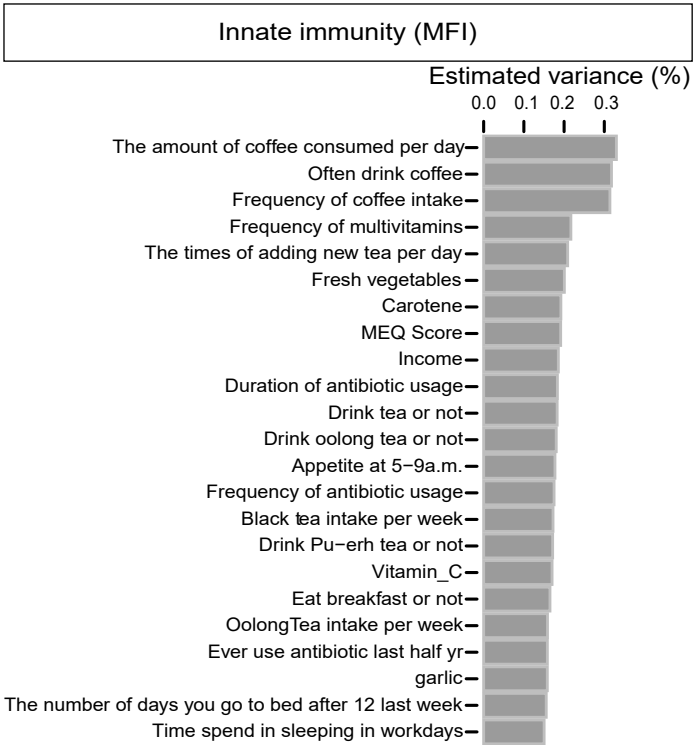

D

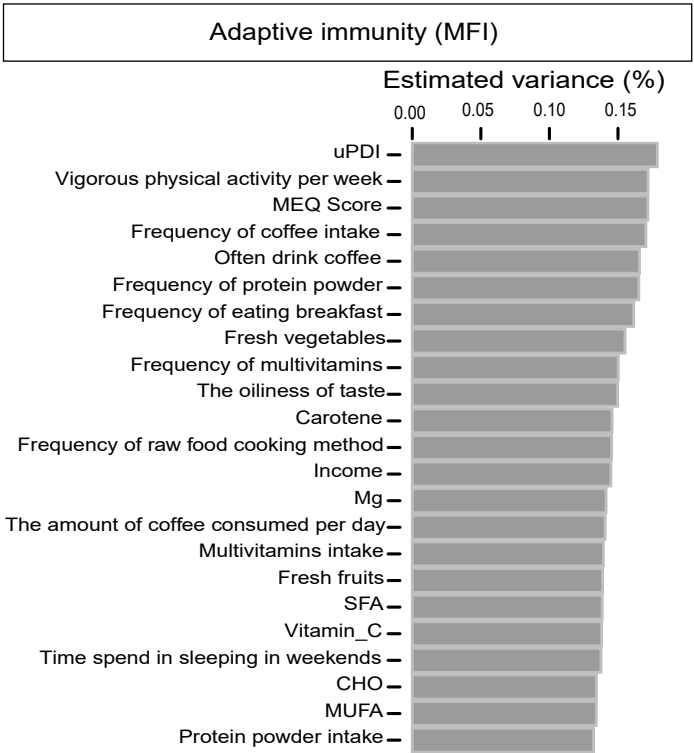

Supplement: Supplementary 1 — Figs. S1 to S14 Tables S1 to S18 Data S1 to S5 [file research.1081.f1.zip › Fig S4.pdf]

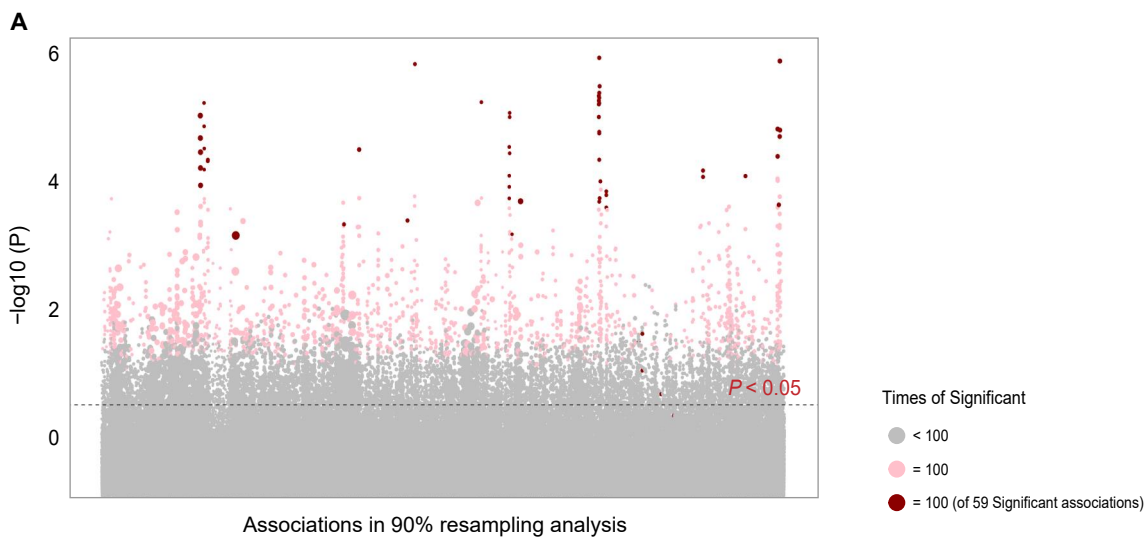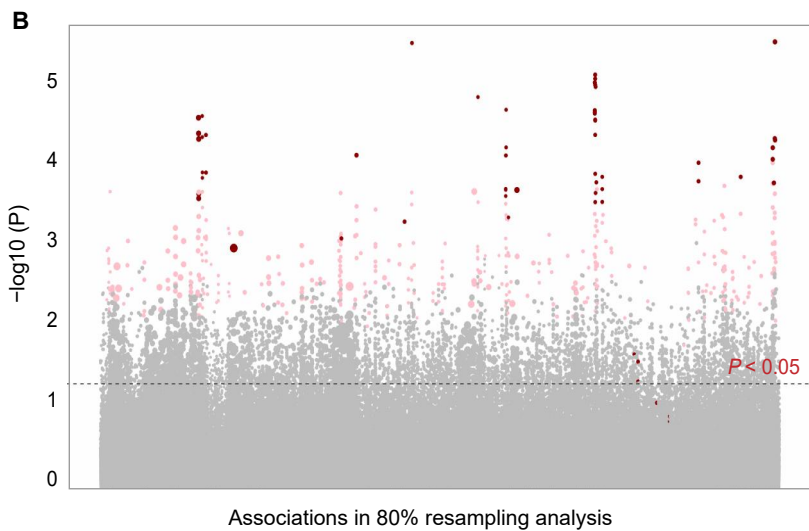

Supplement: Supplementary 1 — Figs. S1 to S14 Tables S1 to S18 Data S1 to S5 [file research.1081.f1.zip › Fig S6.pdf]

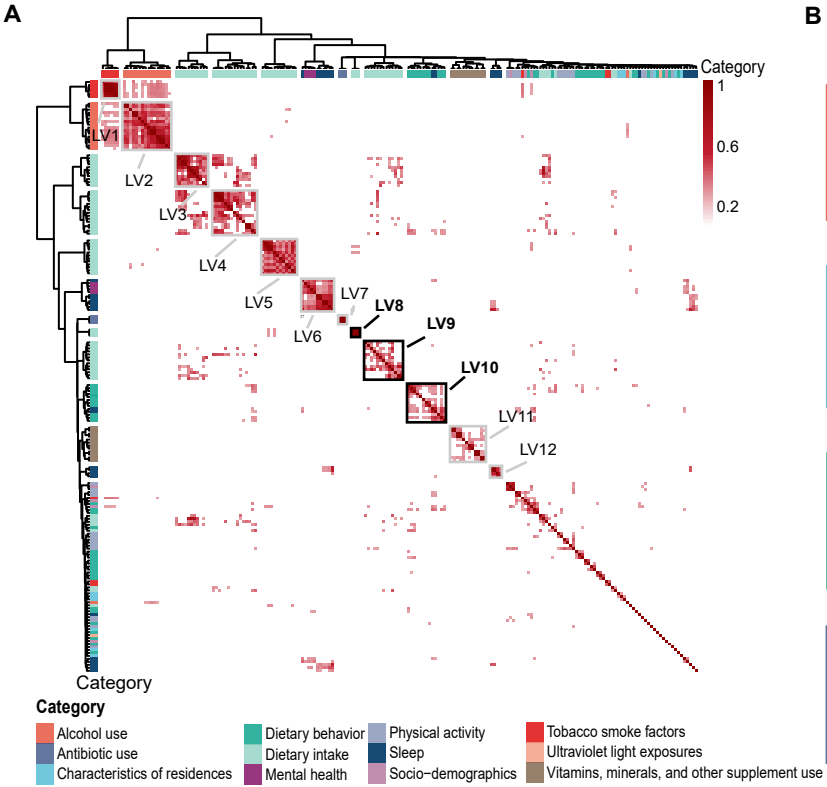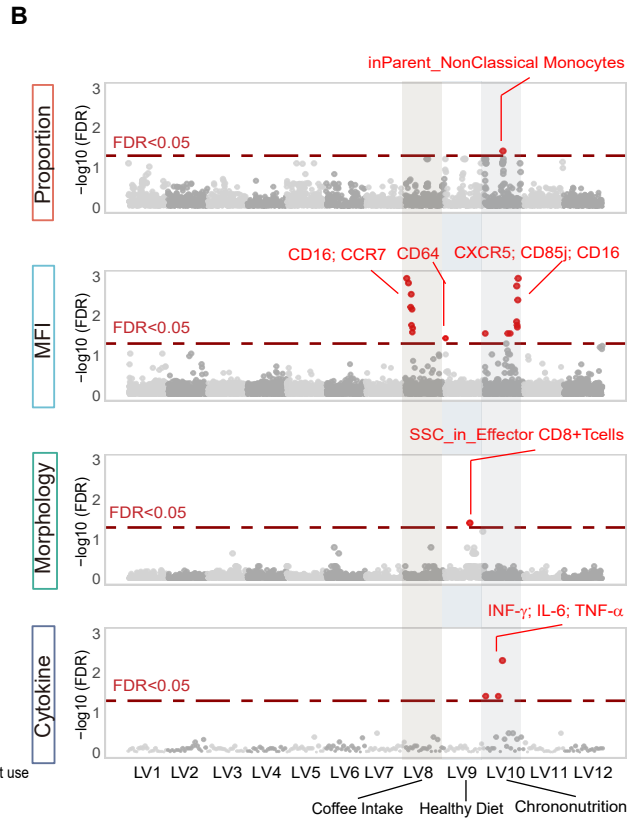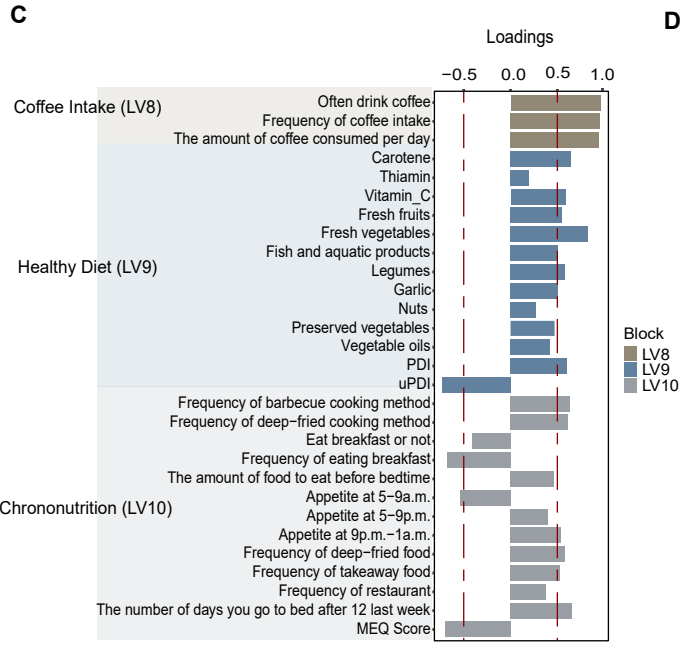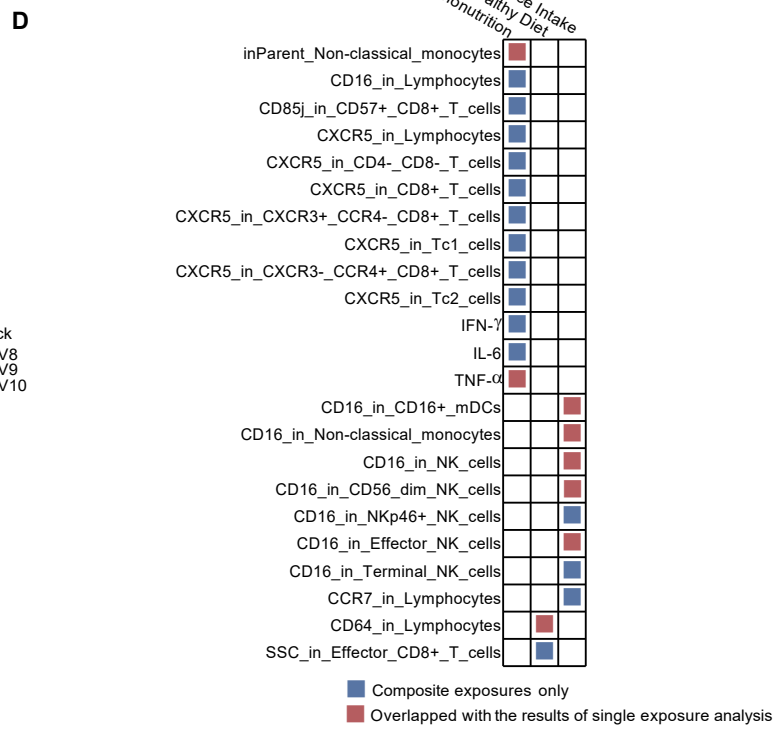

Supplement: Supplementary 1 — Figs. S1 to S14 Tables S1 to S18 Data S1 to S5 [file research.1081.f1.zip › Fig S7.pdf]

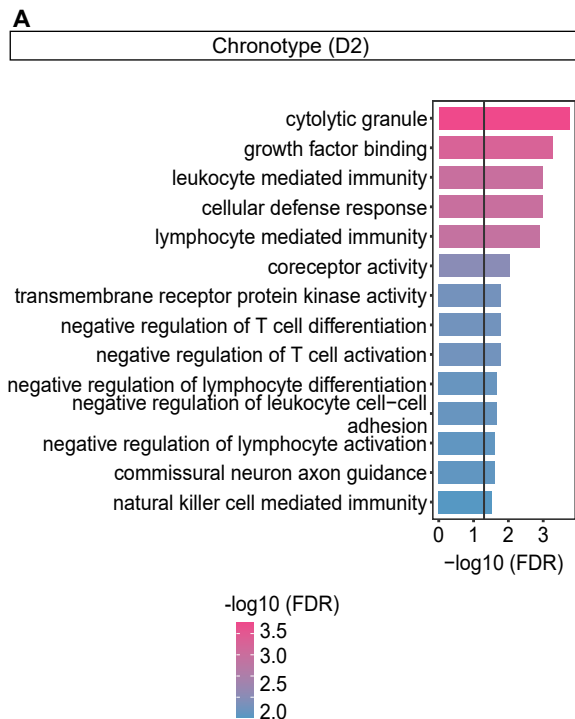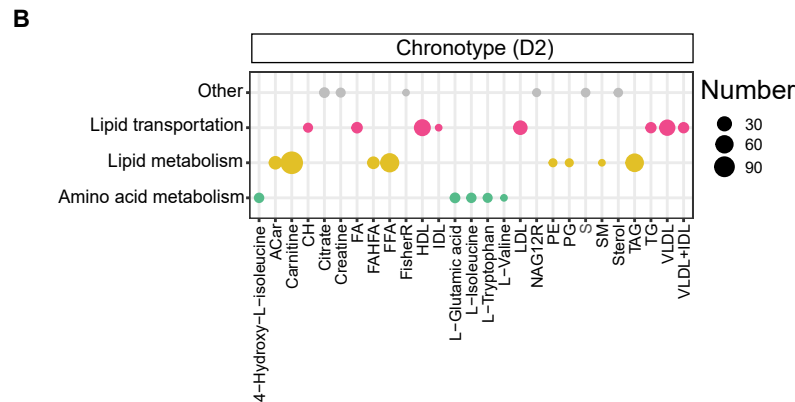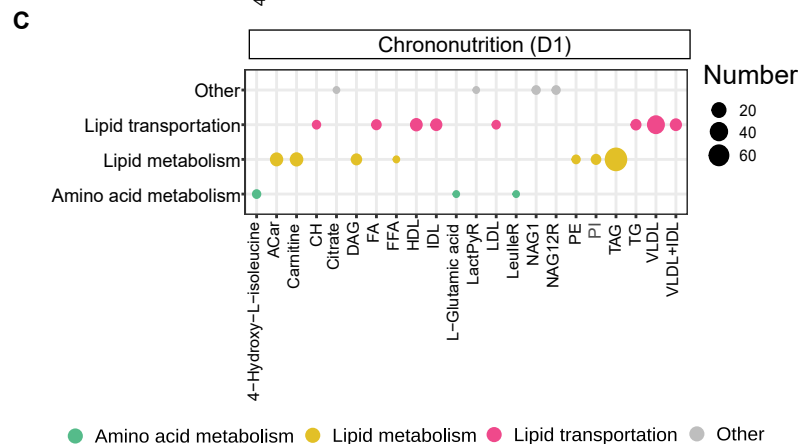

Supplement: Supplementary 1 — Figs. S1 to S14 Tables S1 to S18 Data S1 to S5 [file research.1081.f1.zip › Fig S9.pdf]
